# Biological characteristics and clinical management of uveal and conjunctival melanoma

**DOI:** 10.32604/or.2024.048437

**Published:** 2024-07-17

**Authors:** SNJEŽANA KAŠTELAN, ANA DIDOVIĆ PAVIČIĆ, DARIA PAŠALIĆ, TAMARA NIKUŠEVA-MARTIĆ, SAMIR ČANOVIĆ, PETRA KOVAČEVIĆ, SUZANA KONJEVODA

**Affiliations:** 1 School of Medicine, University of Zagreb, Zagreb, 10000, Croatia; 2 Department of Ophthalmology, Clinical Hospital Dubrava, Zagreb, 10000, Croatia; 3 Department of Ophthalmology, Zadar General Hospital, Zadar, 23000, Croatia; 4 Department of Medical Chemistry, Biochemistry and Clinical Chemistry, School of Medicine, University of Zagreb, Zagreb, 10000, Croatia; 5 Department of Biology and Genetics, School of Medicine, University of Zagreb, Zagreb, 10000, Croatia; 6 School of Medicine, University of Split, Split, 21000, Croatia; 7 Department of Health Studies, University of Zadar, Zadar, 23000, Croatia

**Keywords:** Uveal melanoma, Conjunctival melanoma, Genetic characteristics, Immune checkpoint inhibitors, Target molecular inhibitors

## Abstract

Uveal and conjunctival melanomas are relatively rare tumors; nonetheless, they pose a significant risk of mortality for a large number of affected individuals. The pathogenesis of melanoma at different sites is very similar, however, the prognosis for patients with ocular melanoma remains unfavourable, primarily due to its distinctive genetic profile and tumor microenvironment. Regardless of considerable advances in understanding the genetic characteristics and biological behaviour, the treatment of uveal and conjunctival melanoma remains a formidable challenge. To enhance the prospect of success, collaborative efforts involving medical professionals and researchers in the fields of ocular biology and oncology are essential. Current data show a lack of well-designed randomized clinical trials and limited benefits in current forms of treatment for these tumors. Despite advancements in the development of effective melanoma therapeutic strategies, all current treatments for uveal melanoma (UM) and conjunctival melanoma (CoM) remain unsatisfactory, resulting in a poor long-term prognosis. Ongoing trials offer hope for positive outcomes in advanced and metastatic tumors. A more comprehensive understanding of the genetic and molecular abnormalities involved in the development and progression of ocular melanomas opens the way for the development of personalized therapy, with various potential therapeutic targets currently under consideration. Increased comprehension of the molecular pathogenesis of UM and CoM and their specificities may aid in the development of new and more effective systemic therapeutic agents, with the hope of improving the prognosis for patients with metastatic disease.

## Introduction

Melanocytes, originating from neural crest cells, are distributed throughout the human body in areas including the skin, eyes, cochlea, meninges, and the heart. Additionally, they are present in the mucosal membranes of the respiratory, gastrointestinal, and urogenital tract [1,2]. Their primary function involves synthesizing the melanin pigment within melanosomes. In the epidermis, melanocytes transport melanosomes containing melanin to neighboring keratinocytes, ensuring consistent pigmentation and effective protection against the harmful effects of ultraviolet (UV) radiation from sunlight [3,4]. The function of melanocytes in the skin is well-established, however, their role in other anatomical locations is still not fully understood. The presence of these cells in organs not exposed to UV radiation suggests that they may serve purposes beyond photoprotection [3].

In the eyes, melanocytes are situated in the conjunctiva and various parts of the uvea, including the iris, ciliary body, and choroid [5]. The colour of the eye is determined by the quantity and quality of melanin pigment in the iris and unlike the skin, its colour remains unchanged after sunlight exposure. Additionally, the presence of melanin in uveal melanocytes is thought to contribute to protecting the eyes from age-related macular degeneration and uveal melanoma (UM), whilst the function of melanocytes in the conjunctiva remains unclear [5,6]. The exact mechanism by which melanin provides eye protection is still unknown [6,7].

Conjunctival melanoma (CoM), UM, cutaneous (CM), and mucosal melanoma stem from transformed melanocytes, sharing a common embryonic origin and cellular function, manifesting as highly aggressive forms of cancer. Despite their shared origin, the etiopathogenesis, biological behaviour, treatment, and prognosis of these tumors differ significantly [1,2,8–11]. They exhibit unique patterns of genetic alterations and follow distinct routes and tropisms for metastasis [7,8,12]. Consequently, therapeutic advances in the treatment of CM have not translated into improved clinical outcomes for patients with CoM and UM [3,8] (Table 1). Therefore, an enhanced comprehension of the biological features of these melanomas, coupled with the identification of biomarkers and risk factors, will enable timely diagnosis and optimization of therapeutic approaches. This is pivotal for ensuring long-term survival outcomes.

**Table 1:** Biological and clinical features of melanomas

	Uveal melanomas	Conjunctival melanomas	Cutaneous melanomas
Origin of melanocytes	Stroma of the uveal layer of the eye	The basal layer of the conjunctiva	The basal layer of the epidermis of the skin
Location	Uveal tissue: iris, ciliary body, choroidea	Conjunctiva	The skin
Typical location	Choroidea	Near to corneal limbus	The skin most exposed to the sun
Pigmentation	Vary from amelanotic to very dark	Vary from amelanotic to very dark	Vary from amelanotic to very dark
Mean age of onset	58 years	57.4 years	55.3 years
Incidence	4.9 per million	0.4 per million	153.5 per million
Relative incidence in total cases of melanoma	Approx. 5%	Approx. 1%	Approx. 90%
Incidence trend	Stable	Increasing	Increasing
UV radiation as a risk factor	Unconfirmed	Probable	Confirmed
Mutation burden	Very low	High	High
Predisposing factors	Primarily unknown, Ota nevi, heredity in ~1% of cases (BAP1)	Sun, primary acquired melanosis, conjunctival	Sun, melanocytic nevi, heredity in ~2% of cases (Mostly CDKN2A)
		melanocytic nevi	
Metastatic pathway	Via the bloodstream	Via the lymphatic system and the bloodstream	Via the lymphatic system and the bloodstream
Site of metastasis	Liver (89%)	Lymph nodes (cervical, preauricular, parotid and submandibular)	Skin (13%–38%)
	Lung (29%)		Distant lymph nodes (5%–34%)
	Bones (17%)		
	Skin (12%)	Lungs, liver, skin, brain, adrenals	Distant subcutaneous tissues (32%)
	Lymph nodes (11%)		
			Lung (18%–36%)
			Liver (14%–20%)
			CNS (2%–20%)
			Bone (4%–17%)
Genetic mutations	GNA11 (55%)	BRAF (35%)	BRAF (40%)
		NRAS (20%)	NRAS (20%)
			KIT (<5%)
	GNAQ (40%)	NF1 (14%)	BAP1 (<1%)
	SF3B1 (25%)	KIT (5%)	NF1
	EIF1AX (13%)		CDKN2A
	SRSF2 (4%)		
	BAP1 (38% primary, 84% metastasizing UM)		
Chromosome anomalies	Amplification of 6p, 8q	Amplification of 1q, 3p, 7,17q	Amplification of 1q, 3p, 7,17q
		Loss of 9p, 10, 11, 12q	
	Loss of 3, 1p, 6q		Loss of 9p, 10, 11, 12q
Therapy with immune checkpoint inhibitors	Testing is currently in progress–mostly inefficient results	Anti-CTLA4	Anti-CTLA4
		anti-PD1	
		anti-PDL1	Anti-PD1
		Testing is currently in progress	Anti-PDL1
Therapy with targeted molecular inhibitors	No	Anti-BRAF	Anti-BRAF
		anti-MEK	Anti-MEK
		Testing is currently in progress	

Note: UV: ultraviolet, UM: uveal melanoma, BAP1: BRCA1-associated protein 1; CDKN2A: cyclin-dependent kinase inhibitor 2A; CNS: central nervous system, A11: G protein subunit alpha 11, GNAQ: G protein subunit alpha Q, SF3B1: splicing factor 3B subunit 1, EIF1AX: eukaryotic Translation Initiation Factor 1A X-Linked, SRSF2: serine/arginine-rich splicing factor 2, BRAF: B-raf proto-oncogene, NRAS: N-ras proto-oncogene, NF1: neurofibromin 1, CTLA4: cytotoxic T-lymphocyte-associated antigen 4, PD1: programmed death 1, PDL1: programmed death-ligand 1.

### Clinical characteristics of ocular melanoma

Ocular melanomas represent a subset of uncommon cancers, making up approximately 5% of all melanoma cases [3]. While the incidence of UM has remained stable, the occurrence of CoM has shown an upward trend over the years in the United States (US) and several European countries, reflecting the rising incidence of CM [8,9,11].

Despite these differences, the management of both primary types of ocular melanomas is similar and is largely influenced by various disease factors, including tumor diameter and thickness, degree of extraocular extension, and the presence of metastases. In cases without metastasis, local therapy options for UM include radiation therapy or enucleation. For patients with CoM, initial management revolves around wide local surgical excision, coupled with adjuvant therapies such as brachytherapy or localized chemotherapy. Concerning patient outcomes, the 5-year survival rates for localized UM and CoM are comparable, approximating 70%–80% [3,8,9,11].

### Clinical characteristics of uveal melanoma

UM is the most prevalent primary intraocular malignancy in adults, presenting mainly as choroidal melanoma. The incidence of UM is documented in-5 cases per million in the US and 5–7.4 per million in Europe [13] and predominantly affects individuals of Caucasian descent (97.8%). Individuals predisposed to UM typically exhibit characteristics such as light skin, blonde hair, light irides, the presence of uveal nevi, dysplastic nevi, and oculodermal melanocytosis.

The age of onset of UM ranges from 50 to 80, with a median age of 58 years. UM is a tumor arising from the uveal tissue, and it can originate from any part of the uveal tract. Choroidal melanomas are the most prevalent, followed by melanomas of the iris and ciliary body [11].

UM’s clinical presentation depends upon the tumor’s location and size. It can remain asymptomatic, as reported in some studies, with rates of up to 30%. Symptomatic melanomas often lead to visual symptoms, ranging from mild impairment to complete visual loss due to macular involvement, exudation, or retinal detachment. Lesions may present as unilateral or infrequently bilateral and can be single, multifocal, or diffuse. Bilateral and multifocal lesions are more indicative of potential metastasis [13,14].

Iris melanoma represents a variant with an earlier onset compared to other UMs, typically occurring 10–20 years earlier. In 90% of cases, it can be circumferential, and in 10%, it presents as diffuse [15,16] with the most frequent location being the inferior quadrant. This type of melanoma appears as an elevated iris lesion, exhibiting varying pigmentation and usually remaining asymptomatic. Observable symptoms may include heterochromia (a change in iris color) and corectopia (pupil distortion) while less common manifestations encompass secondary glaucoma, ectropion uveae, and hyphema [17,18]. Diffuse iris melanoma is a rare variant and is often misdiagnosed due to its unique characteristics. It presents as a flat and infiltrative lesion with suspicion often arising when heterochromia and ipsilateral glaucoma are observed. Even more uncommon is melanoma of the trabecular meshwork and anterior chamber angle, known as ring melanoma, usually presenting as refractory glaucoma. The most reliable methods for accurate diagnosis involve gonioscopy and ultrasound biomicroscopy [19].

Ciliary body melanoma can result in pupil abnormalities, lens displacement, localized cataracts, and elevated intraocular pressure. Symptomatic melanomas may manifest with blurred vision, photopsia, floaters, visual field loss, and pain. As previously mentioned, approximately 30% of cases can be asymptomatic. Diagnosis of ciliary body melanoma often occurs when the tumor is large in diameter and thickness as it is located behind the iris and remains invisible during the routine ophthalmological examination [20].

The most common type of UM, choroidal melanoma, usually presents as a subretinal mass with varying pigmentation and often adopts a dome-or mushroom-shaped structure. The dome-shaped presentation is predominantly seen in 75% of cases while the mushroom-shaped presentation occurs in 19% attributed to the rupture of Bruch’s membrane. The diffuse variant is very rare, being found in only 6% of cases [21]. In terms of pigmentation, the lesion can be more or less pigmented in 55% of cases, non-pigmented in 15%, and mixed colour in 30% of cases.

### Treatment of primary uveal melanoma

Local treatment choices depend on factors such as tumor size and location, patient preferences, and existing comorbidities. Enucleation is the most effective treatment option, but it significantly affects the patient’s quality of life. Alternatively, surgical approaches such as tumor exoresection or endoresection can be considered and they may be combined with local radiotherapy to prevent recurrence [11,13,22–24]. Presently, plaque brachytherapy is the most commonly employed local treatment for UM [11,24]. Proton beam radiotherapy and gamma knife radiotherapy are also frequently used, providing an eye-preserving option with effective local control, particularly in the posterior pole. Potential complications of these therapies include an elevated risk of IOP, cataract formation, and optic neuropathy [11,13,22,23,25]. Additionally, other local treatment options, such as transpupillary thermotherapy, photocoagulation, and photodynamic therapy, are often combined with radiation techniques to decrease the risk of metastasis [11,13,26,27].

### Clinical characteristics of conjunctival melanoma

CoM is classified as a mucosal melanoma and shares many similarities with CM [3]. It represents a rare yet potentially devastating condition, comprising 5%–7% of ocular melanomas and 2%–5% of all ocular tumors [28–32]. The incidence of CoM is 0.2–0.8 per million in the Caucasian population and is rarely reported in non-Caucasians [32–34]. Men and women are reported to be equally affected; however, some studies have found a higher incidence in the male population. The incidence of CoM is increasing [31,35,36], following a trend similar to CM. The incidence is higher in older patients, increasing with age, and is rare in individuals younger than 20. The mean age of presentation is 55–56 years of age [37,38].

Conjunctival lesions may precede the onset of CoM; however, in rare cases, the development of a CoM may be preceded by the presence of a conjunctival nevus. It is estimated that only 5% of CoM originates from nevus and that the progression of conjunctival nevus to melanoma is very rare [9,28]. Primary acquired melanosis (PAM) with atypia usually precedes CoM in 53%–75% of melanoma cases, carrying an estimated 13% risk of transformation to melanoma [39]. A notable portion, ranging from 18%–30%, of CoM arises *de novo*, indicating that they do not develop from a preceding conjunctival lesion [40,41].

A typical presentation involves a pigmented lesion near the corneal limbus, although it can manifest at any location on the conjunctiva. The limbal lesion is usually asymptomatic and may appear as a raised plaque, macule, or tumor. It can range from a tiny lesion, measuring less than a millimeter in diameter and thickness to a large tumor on the surface of the eye, exceeding 10 mm in thickness. Other locations of CoM are less common, but when present, they usually indicate a less optimistic prognosis. These locations include the caruncle, plica semilunaris, palpebral conjunctiva, and fornices [28–30]. In 30% of cases, the location can be multifocal [42].

Clinical suspicion of the presence of CoM arises when observing a large tumor, diverse appearance, immobility to the sclera, prominent feeder vessels, and spread onto the cornea [37]. Another important clinical characteristic is the pigmentation of the lesion, which can vary from an amelanotic which is very rare to a light to dark brown and in some cases a highly pigmented, even black lesion. It can manifest as localized disease or spread on the eye surface, affecting the bulbar and palpebral conjunctiva extending over the limbus onto the cornea. Furthermore, it has the potential to infiltrate deeply into the sclera and other underlying structures of the globe as well as the orbital, nasolacrimal tissues, and paranasal sinuses [9,43,44].

Beyond local spreading, both hematogenous and lymphatic dissemination can also occur. The primary indication of spreading typically involves the development of metastases in the regional lymph nodes. Clinical observations suggest that a primary lesion located medially tends to extend toward the submandibular nodes, whereas a lateral location tends to impact the preauricular nodes [9,45]. Hematogenous spreading and distant metastases can occur independently of prior regional disease. The prevalent sites of metastasis encompass the lungs, brain, liver, gastrointestinal system, and skin [11]. The mortality rate for CoM is approximately 30% [36,46].

### Treatment of primary conjunctival melanoma

The optimal strategy for treating all resectable CoM involves complete surgical excision with clear margins, ideally ranging from 2 to 4 mm. In cases of corneal involvement, this procedure can be complemented by additional alcohol corneal epitheliectomy [24,28–30,47]. For cases exhibiting diffuse, lateral, intraepithelial spread, or underlying PAM, the surgical excision is often combined with adjuvant therapies, such as brachytherapy, cryotherapy, and the local application of chemotherapeutic and/or immunotherapeutic agents (mitomycin C and interferon alfa-2-beta). Residual or recurrent PAM is managed with excision, cryotherapy, or the application of topical mitomycin C. Employing a “no-touch” technique during surgery, using fresh, sterile instruments at each step, is essential to minimize the risk of tumor recurrence. Special care should be taken to handle samples minimally, placing them on a paper mount to prevent scrolling or distortions that could impact the accuracy of the diagnosis [11,24,28–30]. Closure is accomplished through primary conjunctival apposition or by employing rotary conjunctival flaps, mucosal grafts from the contralateral eye, buccal mucosa, or amniotic membrane transplants [30]. In certain cases, for reconstructing the fornix, suture deepening or a symblepharon ring may be necessary [24,28–30,47]. Even when histology indicates complete surgical debridement, brachytherapy is recommended to account for the possibility that conventional incisions may have overlooked the deepest part of the tumor. Proton beam radiotherapy is the preferred option for lesions involving the fornix or caruncular area, while modified enucleation is necessary for tumors involving the globe. Orbital externalization is advocated for cases extending into the orbit [24,46].

### Genetics of ocular melanomas

**Figure 1:**
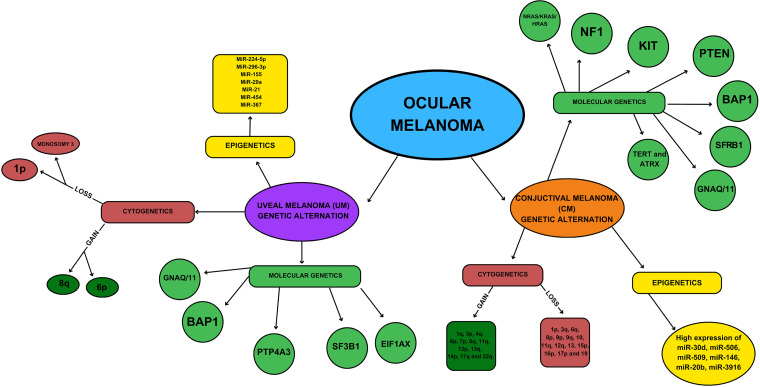
Genetic and epigenetic characteristics of ocular melanoma.

While melanomas occurring in the eye are relatively rare compared to CM, they provide valuable insight into the diverse spectrum of melanoma types present in humans. CoM aligns with epithelium-associated cutaneous and mucosal subtypes, while UM typifies the non-epithelium-associated subtype [48]. Despite the absence of effective therapies for metastatic UM and CoM, recent progress has been made in comprehending the genetics and pathobiology of these malignancies (Fig. 1). The genetic profile of ocular melanoma becomes crucial due to its close association with their metastatic potential. Moreover, the emergence of new therapies, specifically targeted and immune-based, has significantly improved the overall prognosis of the disease [49].

### Genetics of uveal melanoma

#### Genomic alteration in uveal melanoma

UM is characterized by specific genetic mutations, with the most common being mutations in genes like in G protein subunit alpha Q (GNAQ) and G protein subunit alpha 11 (GNA11). These mutations activate pathways related to cell growth and division, contributing to the development of UM. Additionally, mutations in BRCA1-associated protein 1 (BAP1) are associated with a higher risk of metastasis. The activation of the G protein subunit alpha 11/Q (Gα11/Q) pathway plays a crucial role in the early development of UM. This activation is observed in nearly all primary UMs, primarily through single amino acid substitutions GNAQ (57%) and GNA11 (41%) [50]. GNAQ and GNA11 encode closely related G-alpha subunits that are integral components of G protein-coupled receptor proteins (GPCRs). GPCRs are involved in a wide range of physiological functions and play a critical role in tissue homeostasis and cellular proliferation [51]. However, mutations in GNAQ and GNA11 alone are not sufficient for malignant transformation. In cases where primary tumors do not exhibit mutations in GNAQ or GNA11, they often have mutations in genes associated with the Gα11/Q pathway, such as Cysteinyl leukotriene receptor 2 (CYSLTR2) and Phospholipase C β4 (PLCB4). CYSLTR2 encodes a G-protein-coupled receptor that is constitutively activated in 4% of primary UM cases, while PLBC4 activates downstream signaling by directly binding to Gαq and is activated in 2.5%–4% of primary UM cases [52]. Activation of the mitogen-activated protein kinase (MAPK) cascade is observed in up to 86% of primary UM cases [53]. Activation of the MAPK pathway through targets like RAF, MEK, and ERK results in the transcription of genes involved in proliferation, differentiation, and cell survival. MEK inhibitors have been explored as a potential treatment for UM [54]. The PI3K/AKT pathway is highly activated in many cancers and promotes proliferation while reducing apoptosis. Its expression is associated with a poor prognosis in UM. The tumor suppressor Phosphatase and tensin homolog (PTEN) negatively regulates the AKT/protein kinase B (PKB) signaling pathway, and loss of heterozygosity of PTEN markers is observed in 76% of primary UMs. Downregulation of PTEN is suggested to be a late event in tumor progression, linked to increased aneuploidy [55].

The Gαq/11 signaling pathway also promotes the activation of the Trio/Rho/Rac/YAP1 pathway, with YAP hypothesized to promote the transcription of factors linked to cell growth and viability, making it a potential therapeutic target [56].

In addition to mutations in GNAQ, GNA11, PLCB4, and CYSTLR2, UM is distinguished by alterations in three secondary driver genes: BRCA1 associated protein 1 (BAP1), Splicing factor 3B subunit 1 (SF3B1), and Eukaryotic translation initiation factor 1A, X-linked (EIF1AX).

In 2004, Onken et al. introduced the concept of subclustering UM into two well-defined molecular classes using gene expression profiles. This classification yielded two distinct groups: Class 1 (comprising low-grade tumors, with subcategories 1a denoting low-grade tumors and 1b signifying low-grade tumors with metastatic potential) and Class 2 (encompassing high-grade tumors). This division demonstrated a robust correlation with cytological severity and overall survival rates [57].

### Cytogenetic characteristics of uveal melanoma

Chromosomal aberrations play a pivotal role in determining the potential for metastatic spread in UM. Among these aberrations, the loss of chromosome 3 stands out as one of the most significant prognostic markers. Monosomy 3 (M3) is strongly linked to reduced survival rates and is frequently associated with risk factors such as larger tumor size, the presence of epithelioid cell types, and extraocular extension [58].

Additionally, partial deletions of one copy of chromosome 3 and isodisomy are correlated with an increased likelihood of developing metastatic disease [59]. The presence of genetic alterations like gain of 8q (including trisomy 8, isochromosome 8q, and c-Myc gene amplification), when combined with M3, substantially worsens the prognosis. For instance, the five-year disease-specific mortality rate for M3 tumors stands at 40%, but when coupled with 8q gain, it escalates to 66% [60].

The loss of only a portion or the entire chromosome 1 also contributes to poor outcomes, and this is more frequently observed in M3 tumors. Similarly, the loss of chromosome 6q is associated with an unfavourable prognosis. In contrast, the gain of chromosome 6p serves as a predictor for a more favourable prognosis and is rarely observed in conjunction with M3 (approximately 4% coexistence) [61]. Chromosomal abnormalities in the q-arm of chromosome 16 are relatively common in UM, although they do not exhibit a significant association with survival or other cytogenetic and histopathological parameters [62].

### Genetics of conjunctival melanoma

#### Genomic alteration in conjunctival melanoma

Much like in UM, the majority of tumors exhibit mutations in key components of the MAPK pathway. The central actors within the MAPK pathway include RAS, B-raf proto-oncogene (BRAF), MEK, and ERK proteins, serving as intermediaries that relay growth signals from the cell membrane to the nucleus [63]. Predominantly, CoM is driven by mutations in genes such as BRAF, N-ras proto-oncogene (NRAS), c-KIT, and neurofibromin 1 (NF1), which induce the continuous activation of the MAPK pathway. Additionally, mutations in NRAS, c-KIT, and PTEN contribute to the activation of the PI3K/AKT/mTOR signaling pathway [64]. BRAF-mutant CoM exhibits a more aggressive clinical behaviour compared to BRAF wild-type melanomas. These mutations are more prevalent in younger male patients and are linked to an increased likelihood of metastasis, occurring through both lymphatic and vascular routes. Furthermore, clinically, BRAF-mutant conjunctival melanomas are more frequently found on the bulbar conjunctiva than on the palpebral conjunctiva, suggesting a potential role of UV radiation exposure in their development. Additionally, they are associated with deeper invasion [65]. After BRAF, the next most frequently mutated gene within the MAPK pathway is NRAS. NRAS is a member of the RAS kinase family, which includes small guanine nucleotide-binding proteins like HRAS, KRAS, and NRAS. These proteins become activated in response to receptor tyrosine kinases. Importantly, NRAS mutations tend to be mostly mutually exclusive with BRAF mutations [66]. NF1 is another frequently altered gene in CoM, as reported in several studies. NF1 encodes the tumor suppressor protein neurofibromin, which serves as a negative regulator of the MAPK pathway. Consequently, mutations in NF1 lead to increased levels of GTPase activation protein (GAP), triggering RAS signaling and the activation of both the MAPK and PI3K/AKT/mTOR pathways involved in cell proliferation [67]. In cases of CoM, an active PI3K/AKT/mTOR pathway has been associated with a higher mitotic index and greater tumor thickness, both indicating poorer prognoses. Additionally, CoM tend to have a low presence of the tumor suppressor PTEN in their nuclear fraction and the PTEN tumor suppressor’s loss of function can also trigger activation within PI3K/AKT/mTOR cell proliferation pathway [68]. Changes in the epigenetics associated with CoM development encompass mutations occurring in the TERT gene promoter, elevated expression levels of specific chemokine receptors and microRNAs within tumor cells, and alterations in chromosomal copy numbers. Around 5% of CoM showcase a singular TERT promoter mutation. Among these, about 1.4% exhibit either a concurrent BRAF mutation or an NF1 mutation. The majority of TERT mutations involve specific nucleotide changes-typically the UV-related signature alterations C>T or CC>TT [5,69,70].

Chemokines are compact proteins believed to influence the proliferation, invasion, and metastasis of diverse tumors. They’re not only secreted by tumor cells but also by stromal and immune cells, actively participating in modulating the immune response against the tumor [71,72] When the chemokine CXCL12 binds to the chemokine receptor CXCR4, it triggers activation in both the MAPK and the PI3K/AKT/mTOR pathways, extending the survival and promoting the dissemination of tumor cells [73]. In a mouse experimental model, there was a strong correlation between the expression of CXCR4 receptors and the likelihood of metastasis in CoM. Consequently, targeting chemokine receptors could offer extra therapeutic avenues, while alterations in their expression profiles could offer crucial insights into the prognosis and metastatic tendencies of CoM tumors [74].

### Cytogenetic characteristics of conjunctival melanoma

Chromosomal copy number alterations (CNAs) have garnered attention in CoM research, particularly in BRAF/NRAS wild-type tumors. These alterations frequently involve losses in regions like 1p, 3q, 6q, 8p, 9p, 9q, 10, 11q, 12q, 13, 15p, 16p, 17p, and 19, as well as gains in areas such as 1q, 3p, 4q, 6p, 7, 8q, 11q, 12p, 13q, 14p, 17q, and 22q. Among these alterations, a notable commonality across cutaneous, uveal, and conjunctival melanomas is the gain in the 6p region. Loss of the 10q region, housing the PTEN locus, has been associated with lymphatic and metastatic spread, increased tumor thickness, and a mutated BRAF gene. About 30% of BRAF mutations and 43% of NRAS mutations in CoM are attributed to losses or gains in oncogenic loci. Specifically, NRAS mutations often align with gains in 1q, 3p, or 17q, while BRAF mutations.

PRAME (PReferentially expressed Antigen in MElanoma) is a tumor-associated antigen. It was identified on the tumor-reactive T-cell clones derived from a patient with metastatic cutaneous melanoma. PRAME is identified as an important biomarker for metastatic risk in class 1 uveal melanoma and an attractive target for immunotherapy. Recent clinical studies indicate that PRAME expression, in combination with loss of nBAP1, could be a useful predictive biomarker in the therapeutic management of UM patients at high risk [5,64,69].

### miRNA expression uveal melanoma vs. conjunctival melanoma

miRNAs, or small non-coding RNAs, provide a challenge to researchers studying the molecular pathways behind the emergence of malignant illnesses. Specifically, miRNAs affect the regulation of cell proliferation and differentiation because they regulate the expression of mRNA and proteins.

According to the available literature, we can find only scarce data about miRNA profiling in CoM. To the best of our knowledge, only several studies from the same group of investigators represent results of miRNA expression in CoM. These investigations were conducted using patient-archived samples from the Danish Registry of Pathology and the Eye Pathology Institute. In contrast to normal tissues, Larsen et al. [75] found that expression analysis of miRNAs showed overexpression of 24 miRNAs and downregulation of just one. Furthermore, distinct expression patterns were observed for seven miRNAs specific to CoM-tumor stages T1 and T2, which were enhanced and consequently associated with growing tumor thickness. Table 2 lists Hsa-miR in this manner. The expression of miR-3687 and miR-3916 was linked to a higher chance of local recurrence [75]. Through global miRNA profiling of metastatic CoM, the expression patterns of patients with metastases and those without were compared. When comparing main CoM with metastases to CoM without metastatic spread, nine miRNAs showed a substantial downregulation. Six miRNAs were found to be downregulated [35].

**Table 2:** miRNA expression patterns in conjunctival and uveal melanoma: identifying differential expressions

Type of samples investigated	UPREGULATED (↑) or DOWNREGULATED (↓) Hsa-miRNA		Reference
CoM *vs*. normal tissue	↑	miR-20b-5p, miR-128-3p, miR-132-3p, miR-138-5p, miR-146a-5p, miR-146b-5p, miR-181a-5p, miR-181a-2-3p, miR-181b-5p, miR-345-5p, miR-363-5p, miR-500a-5p, miR-500b-3p, miR-501-3p, miR-501-5p, miR-502-3p, miR-506-3p, miR-509-3p, miR-509-3-5p, miR-510-5p, miR-532-3p, miR-532-5p, miR-1260 miR-3689	Larsen et al. [75]
	↓	miR-4689	
CoM stages T1 and T2 *vs*. normal tissue	↑	miR-30d-5p, miR-138-5p, miR-146a-5p, miR-500a-5p, miR-501-3p, miR-501-5p, miR-502-3p	Larsen et al. [75]
Metastatic CM *vs*. nonmetastatic	↑	miR-518a-5p, miR-527, mir-575, mir-622, mir-4501, hmir-4654, mir-4698, miR-6759-5p, miR-8078	Mikkelsen et al. [35]
	↓	miR-34a-3p, mir-548-f4, miR-1270, miR-1290, mir-4278, miR-4528	
UM cell lines or patient tissues *vs*. normal tissue	↑	miR-21, miR-155, miR-181, miR-181b miR-367, miR-454 miR-652	Pasalic et al. [10]
	↓	miR-9, miR-17-3p, miR-34a, miR-122, miR-124a, miR-137, miR-142-3p, miR-144, miR-145, miR-182, miR-205, miR-216a-5p, miR-224-5p	
High metastatic tissue samples *vs*. primary tumors	↑	let7b, miR-miR-199a-3p/5p, miR-143, miR-193b, miR-652	Worley et al. [76]
Tumors with M3 or D3 (normal melanocytes)	↑	miR-134, miR-146b*, miR-149, miR-199a, miR-214 miR-1238,	Venkatesan et al. [80]
	↓	let-7b, miR-143*, miR-146b**, miR-199a	
High-risk UM metastasis *vs*. low-risk patients	↑	miR-16-5p, miR-17-5p, miR-21-5p, miR-132-5p, miR-151a-3p,	Smit et al. [82]
	↓	miR-let-7c-5p, miR-99a-5p, miR-99a-3p, miR-101-3p, miR-miR-181b-5p, miR-101-3p, miR--2-3p, miR-378d	
Metastatic UM *vs*. nonmetastatic UM patients	↑	miR-346, miR-592, miR-1247	Wroblewska et al. [88]
	↓	miR-506, miR-513c	
High *vs*. low-grade tumor stage	↑	miR-592 and miR-199a-5p	Falzone et al. [85]
	↓	miR-508-3p, miR-509-3-5p, miR-513c-5p, and miR-513a-5p, miR-514a-3p,	
Metastatic UM *vs*. nonmetastatic UM patients	↑	miR-199a-5p, miR-592, miR-708-5p	Vashishtha et al. [87]
	↓	miR-506-3p, miR-508-3p, miR-508-5p, miR-509-3p, miR-509-3-5p, miR-513c-5p, miR-513a-5p, and miR-513b-5p, miR-514a-3p	
Metastatic UM *vs*. nonmetastatic UM patients	↑	miR-624	Triozzi et al. [86]
	↓	miR-506, miR-508-3p, miR-509-3-5p, miR-509-3p, miR-513a-5p, miR-513b, miR-935	
Metastatic UM *vs*. nonmetastatic UM patients	↑	miR-16, miR-145, miR-146a, miR-204, miR-211, and miR-363-3p	Worley et al. [76]
UM patients *vs*. healthy controls	↑	miRNA-146a, miR-523	Russo et al. [79]
	↓	miR-19a, miR-30d, miR-127, miR-451, miR-518f, miR-1274b	

Note: *Downregulated only in D3 (with or without metastasis). **Downregulated only in D3 UM with liver metastasis, upregulated in D3 without liver metastasis and in M3 (with and without liver metastasis). M3_ monosomy 3, DM-disomy 3. ^#^Bolded miRNA recognised in CoM and UM.

In contrast to CM, there is a greater amount of data associated with studies on miRNA expression in UM. Our earlier literature review included comprehensive findings from many studies that examined the function and variations in miRNA expression in UM [10].

Upon reviewing the literature, we discovered that some miRNAs known to function as tumor suppressors had lower expression levels in UM cell lines or patient tissues in comparison to normal tissue. Conversely, oncogenic miRNAs were found to be increased in UM tissues or cell lines. Table 2 displays the expression of miRNA in several human CM and UM samples, with at least one miRNA revealing distinct expressions in both CM and UM [10,35,76].

According to the data represented in Table 2, it seems that UM and CoM exhibit partially different lists of upregulated and downregulated miRNA. This indicates that miRNA profiling might be useful in the differentiation between those two ocular melanomas. As far as we know and based on the literature reviewed in this article, seven miRNAs were shown to have altered expression in both CM and UM. They are listed as follows: miR-146a-5p, miR-146b-5p, miR-181b-5p, miR-363-5p, miR-506-3p, miR-509-3p and miR-509-3-5p.

It was observed that the expression of miR-146a increased in advanced stages of tumors and that it improved in both CM and UM ocular melanoma [75,77]. Depending on the type and location of the cancer, this miRNA may perform the functions of an oncomiR, a tumor suppressor, or both. It directly targets genes in different types of malignancies [78]. Various studies verified that miR-146a can additionally promote the migration and invasion of malignant melanoma and demonstrated a correlation between miR-146a levels and melanoma recurrence [78,79].

When compared to normal tissue, miR-146b-5p increased in CoM patients, whereas in UM patients, it was upregulated in tumors with M3 or D3 monosomy when compared to normal melanocytes [75,80]. One of the miRNAs that were upregulated in cutaneous melanoma was miR-146b-5p, according to a comparative microarray investigation of the microRNA expression profiles in primary cutaneous malignant melanoma [81]. These findings suggest that miR-146b-5p functions as an oncomiR in several melanoma subtypes.

Compared to normal tissue, the tissues of CoM patients have higher levels of miR-181b-5p [75]. Various investigations on UM cell lines or tissues from UM patients also revealed elevation of miR-181b-5p relative to normal tissue [10]. According to a study by Smith et al., patients with high-risk UM metastases had lower levels of the same miRNA than patients at low risk [82]. Certain isomiR variations exhibit an opposite pattern in cases of cutaneous melanoma. As a result, it was determined that one miR-181b-5p had at least two variants with expression patterns that were in opposition to one another [83].

miR-363-5p that was upregulated in CoM patients [75] showed to be upregulated in patients with metastatic UM [76]. Melanocytic Spitz tumors exhibited higher expression of miR-363-3p in comparison to benign nevi, melanoma cell lines, and ulcerated *vs*. non-ulcerated melanoma [84].

Investigation revealed that, in contrast to individuals with non-metastatic tumors, patients with metastatic UM tumors had downregulated levels of the miRNA miR-506-3p, which was upregulated in CoM [75,85–88]. The tumor suppressor effect of miR-506 was indicated by its substantial downregulation in highly invasive Mel Im cell-melanoma metastases [89].

MiR-509-3p and miR-509-3-5p, both were downregulated in high-grade tumor stage and metastatic UM. Therefore, they might be considered to act on suppressing tumors. In CM both miRNAs showed increased expression in tumor tissue than in a health sample [90]. A comprehensive review of the scientific literature revealed that miR-509-3-5p has not yet been linked to any other forms of melanoma [91].

We predict that the above-mentioned miRNAs may be taken into consideration and researched as potential therapeutic targets. Additional research on other populations and validation of miRNAs that have been demonstrated to be differentially expressed may prove beneficial in the future for differential diagnosis.

### Similarities and differences of additional epigenetic mechanisms between conjunctival and uveal melanoma

The most thorough analysis revealed unique epigenetic characteristics for UM, although the DNA methylation profile of CM exhibited strong similarities to mucosal and cutaneous melanoma [92]. In conjunctival melanoma, hypermethylation was seen in the promotor region of the APC gene and the CDKN2A promoter area linked to the p16 transcript. RASSF1 promoter hypermethylation was reported for uveal melanoma [92].

When compared to normal uvea, UM delta-like protein 3 (DLL3) overexpression demonstrated that all of the CpGs of DLL3 were hypermethylated in uveal melanoma tissues [93]. Compared to the metastatic UM samples, the non-metastatic UM samples had a considerably higher expression level of DLL3 [93]. This most likely indicates the DLL3 gene’s protective role. Additionally, there was a considerable increase in DLL3 expression in the CM samples compared to the healthy ocular tissues [93].

A relationship exists between SF3B1 and BAP1 mutations and metastatic UM. According to Smith et al. UM metastasis mediated by SF3B1 and BAP1 is affected by aberrant methylation [94]. Additionally, they revealed aberrant DNA methylation in some of the new genes that are correlated with modifications in gene expression in primary UM and its metastases, including KLF10, GSTP1, and MEGF10 [94].

Histone acetylation and deacetylation play pivotal roles in regulating gene expression by modifying chromatin structure. Specific histone modifications like monomethylation of H4K20 and H2BK5, as well as trimethylation of H3K4, H3K36, and H3K79, tend to promote gene expression. Conversely, dimethylation of H3K9 and trimethylation of H3K9 and H3K27 usually inhibit gene expression [95].

Holling et al. found a connection in UM cells between reduced CIITA transcript levels and an elevated occurrence of trimethylated histone H3-lysine 27 (H3K27me3) within CIITA-PIV chromatin, rather than DNA methylation of MHC2TA promoter IV (CIITA-PIV). Their study showcased the presence of EZH2, a histone methyltransferase, within CIITA-PIV chromatin. EZH2 is recognized as part of the polycomb repressive complex 2 and can add three methyl groups to histone H3-lysine 27 [96]. Additionally, UM cell lines exhibiting an overexpression of the HES1 transcription factor are linked to their metastatic potential. One contributing factor to this heightened expression of HES1 in UM cells is the H3K4 trimethylation of the HES1 promoter [97].

Research indicates that reduced BAP1 expression resulted in compromised differentiation among UM cells. However, this crucial impairment can be partially alleviated by HDAC inhibitors, known to enhance histone H3 acetylation [98]. In conjunctival melanoma, histone acetylation and deacetylation dynamics can influence the expression of genes involved in various cellular processes, potentially impacting tumor progression. In conjunctival melanoma, specific studies elucidating the precise role of histone acetylation and deacetylation mechanisms might still be evolving.

### Immune checkpoint inhibitors in ocular melanoma therapy

Immune checkpoint inhibitors (ICIs) have become integral in treating various cancers, notably advanced melanoma and non-small cell lung cancer. Some tumors utilize mechanisms to elude immune surveillance by triggering T-cell checkpoints. Activating these checkpoints allows tumors to escape the cellular immune response, facilitating their survival and dissemination. ICIs play a pivotal role in thwarting the activation of these checkpoints, effectively stimulating the immune system and strengthening the antitumor response. The most extensively researched inhibitory checkpoint pathway includes cytotoxic T lymphocyte-associated molecule-4 (CTLA-4), programmed cell death receptor-1 (PD-1), and programmed cell death ligand-1 (PD-L1) [99]. The introduction of immunotherapy in treating CM has significantly improved the prognosis of these patients [99,100]. The first approved monoclonal antibody was Ipilimumab, an antibody targeting CTLA-4, followed by Nivolumab and Pembrolizumab, monoclonal antibodies that target PD-1 and Atezolizumab, Durvalumab, and Avelumab target PD-L1 [100].

The concept of the eye’s immune privilege represents a mechanism, conferring the ability to shield itself from uncontrolled inflammation and preserve vision. Additionally, this phenomenon plays a role in how UM manages to evade the expected immune response to tumor cells in other locations [101,102]. The blood-eye barrier serves as a protective mechanism, limiting the entry of inflammatory cells into the eye from the bloodstream. Key immunoregulatory components in this process include PD-L1 and indoleamine 2,3-dioxygenase (IDO). Its primary function is to protect non-regenerating ocular tissues. Within the anterior chamber and aqueous humour, numerous cytokines with anti-inflammatory and immunosuppressive properties are present, including transforming growth factor-beta (TGF-β), complementary regulatory protein (CRP), macrophage migration inhibitory factor (MIF), vasoactive intestinal peptide (VIP), α-melanocyte-stimulating hormone (α-MSH), and a low expression of major histocompatibility complex (MHC) [101,103]. Additional mechanisms include the suppression of T cell proliferation, a decrease in MHC class expression in the cells, and the expression of PD-L1 and PD-L2 on the retinal pigment epithelial (RPE) cells, inhibiting T cell responses [104,105].

Among its various functions, the immune system plays a crucial role in regulating and controlling tumor growth and progression. Tumor tissue seeks to evade immune destruction through alterations in its immunogenetics and microenvironment (TIM). The immune process is a finely regulated mechanism aimed at preventing an excessive immune reaction that may result in tissue damage, with ICIs serving as a key mechanism for maintaining this control [106].

### Immune checkpoint inhibitors for uveal melanoma

Immunotherapy utilizes the antitumor potential of ICIs such as CTLA-4 and PD-1 along with their ligands B7 and PD-L1 [107,108]. These ICIs act as antibodies, by binding to checkpoints and suppressing them. Consequently, T cells are activated and proliferated, leading to the destruction of tumor cells [109]. Monoclonal antibodies targeting CTLA-4 and PD-1, like Ipilimumab, enable suppressed antitumor immune regulators to respond by blocking the immune response to tumor antigens [101,110]. This activation of the immune system results in tumor regression [111]. Currently, key agents employed in the immunotherapy of UM include Ipilimumab (anti-CTLA-4), Nivolumab and Pembrolizumab (anti-PD-1), and Atezolizumab, Durvalumab, and Avelumab (anti-PD-L1) [99,100]. Numerous clinical studies on immunotherapy in UM have been conducted, encompassing patients at different clinical stages of the disease. Regrettably, the therapy’s results have not met the anticipated level of success and do not align with outcomes seen in other primary melanomas, particularly in CM and CoM [112].

In the studies conducted by Zimmer et al. [113–115], the investigation focused on the efficacy and safety of Ipilimumab administered at a dose of 3 mg/kg every three weeks for four cycles. The overall survival (OS) rates were 6.8, 6, and 5.2 months, respectively, while the median progression-free survival (PFS) was 2.8, 3.6, and 2.9 months, respectively. The safety profile of Ipilimumab in these studies was comparable to that observed in patients with CM. Treatment-related grade 3–4 adverse events (AEs) were reported in 36%, 6%, and 13.6% of cases, respectively. Notably, none of the patients in these studies achieved a complete response (CR) to the therapy. A retrospective analysis investigating the administration of Ipilimumab in Europe and the USA, conducted by Luke et al. [116], at higher doses of 10 mg/kg every three weeks, showed a response rate of 2.6% at 12 and 23 weeks with AEs of grades 3 and 4 in 17.9% of patients. The median OS was 9.6 months from the first dose. The findings from these studies and retrospective analyses suggest that anti-CTLA-4 antibodies exhibit limited effectiveness in metastatic UM. However, in some patients, there is an observed improvement in OS compared to standard chemotherapy.

The effectiveness of Nivolumab and Pembrolizumab, both anti-PD-1 receptor antibodies, was examined to assess their potential in treating UM. Insights gained from trials conducted by Kottschade et al. [117–119] suggest that agents targeting anti-CTLA-4, anti-PD-1, and anti-PD-L1 pathways did not exhibit promising efficacy in UM treatment. The mentioned investigations underscore a lack of substantial therapeutic benefit from these agents in the context of UM. The outcomes from conducted studies challenge the initial expectations regarding the potential of these immunotherapies in treating UM, emphasizing the need for further exploration and alternative therapeutic approaches for this specific malignancy.

Some studies were conducted using combined therapy, namely Schadendorf et al. [120] and Heppt et al. [121] performed comparisons between monotherapy and combination therapy using two antibodies. Their findings revealed that the response to anti-PD1 agents is significantly more favourable for lung and skin metastases compared to other organs. Recent studies highlight the importance of PD-L1 expression on melanoma cells, revealing a significantly lower presence in metastatic UM compared to metastatic CoM cells [122,123]. Moreover, there is an indication of site-specific expression, with cells in liver metastases displaying even lower PD-L1 levels. This suggests an additional pathway through which metastatic UM may elude the immune system. The diminished PD-L1 expression in metastatic UM potentially explains the limited efficacy observed in PD-1 inhibitor therapy, implying immune evasion occurring through this pathway [124]. Table 3 represents the data of anti-CTLA-4 and anti-PD-1 treatment efficacy depending on the PD-L1 expression on the metastatic melanoma cells.

**Table 3:** Immune checkpoint inhibitors in ocular melanoma therapy

	Conjunctival melanoma	Uveal melanoma
PD-L1 expression on metastatic melanoma cells	High	Low
Agent	Anti-CTLA-4 Ipilimumab	Anti-CTLA-4 Ipilimumab
	Anti-PD-1 Pembrolizumab	
		Anti-PD-1 Pembrolizumab, Nivolumab
		Anti-PD-L1 Atezolizumab, Durvalumab, Avelumab
Treatment outcome	Partial or complete response	Low overall response rate, prolonged overall survival
Combined therapy	Synergistic action	Low-rate results, mild synergism

Note: CTLA4; cytotoxic T-lymphocyte-associated antigen 4, PD1; programmed death 1, PDL1; programmed death-ligand 1.

Another significant area of investigation involves the prolonged latency period between the treatment of the primary tumor and the onset of metastatic disease. Tumor cells can persist in a dormant state during this period which may be linked to the primary site of the tumor namely the eye, known for its immunosuppressive environment. Consequently, there is potential for new therapeutic investigations to focus on targeting pathways associated with this dormancy [125].

Lymphocyte activation gene-3 (LAG-3) is a co-inhibitory receptor on T cells. It suppresses their activation. It is a promising agent in immunotherapy melanoma. LAG-3 inhibitors combined with PD-1 inhibition have better efficacy and similar toxicity in comparison to single-agent PD-1 inhibitors. This combination has been approved by the U.S. Food and Drug Administration as the first-line therapy for patients with metastatic melanoma [125,126].

### Immune checkpoint inhibitors for conjunctival melanoma

The existence of molecular similarities between CM and CoM, along with the expression of PD-1/PD-L1 in a subset of CoM, implies the potential significance of checkpoint inhibition as a viable treatment option for CoM [9]. However, there is limited data with only a few cases reports and case series discussing the use of ICIs for recurrent, locally advanced, and metastatic CoM [127].

However, in the area of CoM therapy, ICIs have demonstrated more favorable outcomes compared to their use in UM. Results ranged from complete regression to partial response, with a singular case showing partial response followed by progression. These inhibitors have been effectively applied in both metastatic and locally advanced diseases. The therapeutic approach encompasses single-agent use, sequential therapy, and combination immunotherapy, including novel combinations with intralesional interferon α2b [99,128–131]. Nivolumab and Pembrolizumab, both anti-PD-1 receptor antibodies, act by inhibiting the interaction between PD-1 and PD-L1 which leads to the lysis of tumor cells. Approved for CoM treatment in the US, these agents demonstrate superior efficacy compared to the anti-CTLA-4 agent Ipilimumab [132]. Furthermore, the combined therapy involving anti-CTLA-4 and anti-PD-1 agents shows a synergistic effect, yielding overall improved outcomes in CoM therapy through the downregulation of various phases of T-cell activation. While initial studies showed promising outcomes, the available data on ICIs in CoM is confined to case reports and case series, with no larger controlled trials conducted at present [127].

The challenge associated with immune checkpoint inhibitors arises from the significant side effects triggered by immune system activation. Commonly reported side effects involve the worsening of autoimmune diseases [133,134] while ocular complications may manifest as dry eyes, conjunctivitis, episcleritis, keratitis, uveitis, and orbital inflammation [135,136]. Additionally, varying in severity, other observed side effects impact different organs, including colitis, cholangitis, pancreatitis, hepatitis, adrenal insufficiency, hypothyroidism, type I diabetes, myocarditis, pneumonitis, and acute kidney injury [137–141].

### Targeted molecular inhibitors in ocular melanoma therapy

Over the past decade, targeted therapies or molecularly targeted therapies for malignant tumors have become an important aspect of cancer treatment [28,29]. Operating at the molecular level this form of therapy disrupts the growth of cancer cells by selectively interfering with essential molecules crucial for carcinogenesis and tumor development. In contrast to conventional chemotherapy which affects all rapidly dividing cells throughout the body, targeted molecular therapy offers a more precise approach. Within cancer pharmacotherapy, targeted molecular therapy is one of the three primary strategies, alongside hormonal therapy and cytotoxic chemotherapy. By employing highly specific molecules that singularly impact cancer-related processes this focused treatment approach significantly mitigates side effects compared to the more systemic effects of traditional cancer drugs. Simultaneously, the precision and efficacy of targeting tumor cells may contribute to improved treatment outcomes [142].

### Targeted molecular inhibitors for uveal melanoma

A better understanding of mutations in CM and UM, particularly those associated with the mitogen-activated protein kinase pathway, has paved the way for the development of targeted therapies. The forefront of these advancements lies in the targeted blockade of growth regulatory signaling pathways. Recently, agents designed to inhibit components of the MAPK pathway, such as BRAF and NRAS, have been introduced, representing a significant stride in the advanced treatment of CM. These treatments markedly increased the lifespan of individuals dealing with CM. Regrettably, this success has not been replicated in the case of UM. The key difference between CM and UM lies in the mutational load, with UM showing a low compared to the high mutational load observed in CM [143]. Table 4 represents mutational load, mutation location, targeting driver mutations, and agents in uveal melanomas.

**Table 4:** Targeted molecular inhibitors for ocular melanoma therapy

	Conjunctival melanomas	Uveal melanomas
Mutational load	High	Low
Mutation location	BRAF	GNAQ/GNA11
	MEK	
Targeting driver mutations	BRAF inhibitors	GNAQ11 mutations
	BRAF/MEK inhibitors	Gα mutations
Agents	BRAF inhibitors	siRNA
	Vemurafenib, Dabrafenib	Downregulating GNAQ mutant expression
		FR900359
	MEK inhibitor	
		Targeting wild-type GαQ
	Trametinib	FR900359
		Inhibits GαQ/11/14
Other targets		Inhibition of signaling pathways
		Chromatin structures and transcription
		Hyper-Expressed Molecules Involved in Progression
		Anti-angiogenetic agent

Note: GNA11; G protein subunit alpha 11, GNAQ; G protein subunit alpha Q, BRAF; B-raf proto-oncogene.

UM exhibits mutations in the GNAQ/GNA11 genes in about 90% of cases [144]. The catalytic glutamine is substituted by leucine in the GNAQ/GNA11 Q209L mutant proteins, a presence observed in the early stages of tumor initiation and maintained throughout any stage of UM development [126,145]. This mutation leads to the loss of Guanosine Triphosphate hydrolase (GTPase) activity. Notably, the GNA11 mutation is more frequently identified in metastatic UM compared to the GNAQ mutation [50]. Gα proteins and downstream signaling molecules emerge as potential targets for therapy, given their pivotal role in activating the PLCα/PKC pathway and multiple downstream signaling pathways. Additionally, alternative driver mutations involve genes for phospholipase C4 (PLCB4) [51] and the G protein-coupled receptor (GPCR) cysteinyl leukotriene receptor-2 (CYSLTR2) [146]. These insights provide a comprehensive understanding of the genetic landscape in UM, offering potential avenues for targeted therapeutic interventions.

Molecular therapies for UM primarily target GNAQ/11 mutations, with ongoing developments in addressing mutated Gα. Specific short interfering RNA (siRNA) effectively downregulates GNAQ mutant expression, leading to a reduction in the GαQ protein levels within UM cell lines [147]. This siRNA inhibits the viability and growth of UM cells and can be delivered through innovative methods such as oncolytic viruses [148] and functionalized gold nanoparticles. Distinguishing between the GαQ-Q209L and GαQ-Q209L mutants reveals disparate molecular characteristics. The former, resembling the wild-type protein, demonstrates limited therapeutic potential. In contrast, the latter, by modifying its interaction with Gβγ and regulators of G-protein signaling, holds significant therapeutic promise [149].

Research into the therapeutic potential of a molecule, FR900359, and its derivative, cyclic depsipeptides has yielded promising results. This compound, derived from the plant *Ardisia crenata* Sims, exhibits a high affinity for and acts as a pseudo-irreversible inhibitor of the wild-type GαQ and has demonstrated impacts on cell proliferation and migration [150]. In a recent study, FR900359 was found to inhibit downstream signaling, and cell proliferation, induce melanocytic differentiation, and hinder oncogenic signaling in UM cells [151].

An objective of targeted therapy involves the inhibition of signaling pathways in tumor development. Several signaling pathways are activated downstream of mutation-activated GαQ and Gα11 and are potential targets for therapy. Notably, oncogenic GαQ signaling relies on small GTPase and ADP-ribosylation factor 6 (ARF6), making them viable considerations as potential targets for UM therapy [152]. There are several signaling pathway inhibitors currently in early preclinical studies, such as selumetinib (AZD6244, ARRY-142886) [153] and TAK-733 [154], which demonstrate inhibition of UM cell proliferation and viability *in vitro*. Ongoing studies are also investigating the potential impact of higher doses of selumetinib to more efficiently block the MAPK pathway and prevent resistance development. Initial findings from studies on selumetinib revealed an improvement in the response rate (14% *vs*. 0%) and progression-free survival (PFS) (15.9 *vs*. 7 weeks) in the selumetinib arm compared to chemotherapy. However, the impact on OS was limited (11.8 *vs*. 9.1 months) [155].

Investigations into combined therapy involving MEK inhibitors with other drugs have not demonstrated significant improvements and some combinations are still under investigation. Additionally, other drugs undergoing preclinical investigation include the pan-PKC inhibitor sotrastaurin (AEB071) [156], along with its combinations with MEK inhibitors, showing potential synergistic activity. The pan-PI3K inhibitor is also under investigation, showing potential synergistic action with other inhibitors of downstream pathways, particularly promising in the treatment of UM in a GNAQ-mutated xenograft model [157]. Further, mTOR emerges as a promising therapeutic target, with ongoing investigations into its inhibitor, everolimus, in combination with pasireotide, a somatostatin receptor agonist known for its limited therapeutic potential [158]. Likewise, everolimus is undergoing scrutiny in combination with inhibitors of PKC, MEK, AKT, and PI3K. In *in vitro* models, this combination has shown promising pro-apoptotic effects [159].

The key mediator of oncogenic activity in UM development is the mutation-activated GαQ or Gα11, which induces YAP/TAZ dephosphorylation and signaling. Studies indicate that YAP is a promising target for the therapy of UM with mutations in GNAQ or GNA11 genes. The knockdown of mutated GαQ results in decreased nuclear localization of YAP and its interaction with transcription factors crucial for YAP-mediated processes such as proliferation, epithelial–mesenchymal transition, and oncogenesis [56,160,161]. Furthermore, focal adhesion kinase (FAK) plays a pivotal role in YAP activation in UM cell growth. Current investigations are focused on blocking FAK activity as a potential therapeutic strategy [162].

An additional class of drugs focuses on chromatin structures and transcription, recognizing the crucial role of transcriptional programs in mediating tumor progression [163]. Histone deacetylases (HDAC) are enzymes responsible for epigenetic modifications in cancer cells [164]. HDAC inhibitors investigated in UM *in vitro* show antitumor activities, with some demonstrating the ability to induce a transition from a high-risk to a low-risk gene expression profile [165]. The epidermal growth factor receptor (EGFR) is frequently overexpressed in UM cells, leading to the investigation of its inhibition in combination with other drugs for UM therapy [166].

DNA methylation serves as a target for epigenetic therapy with ongoing research on drugs that inhibit DNA methyltransferase I and facilitate the hypomethylation of DNA, such as azacitidine and similar agents. Understanding the DNA methylation status is essential as it may play a crucial role in modulating the metastatic potential of UM [167]. Targeting hyper-expressed molecules involved in progression is another key aspect of targeted therapy for UM. Ongoing studies are exploring genes that exhibit overexpression in melanoma cells with metastatic potential. Among these genes, the SDCBP gene, responsible for encoding mda-9/syntenin, emerges as pivotal for UM cell migration, invasion, and FAK activation [168]. Elevated expression of several matrix metalloproteinases (MMPs) is detected in primary UM, correlating with a poorer prognosis [169,170]. The inhibition of MMPs has demonstrated efficacy in reducing the invasiveness of UM [171]. Liver metastases of UM strongly express c-Met, the tyrosine kinase receptor. Crizotinib, acting as a c-Met inhibitor, is presently being investigated as a potential adjuvant therapy for metastatic UM, showing promise in reducing metastasis formation *in vitro* [172,173].

Anti-angiogenic agents are currently used in the treatment of various malignancies, often in combination with other therapies, and UM is considered a potential target for such interventions [174]. The concentration of vascular endothelial growth factor (VEGF) is elevated in the aqueous humor of patients with UM and the serum of those with metastatic disease [175,176]. Bevacizumab, the most widely used anti-VEGF agent, has demonstrated efficacy in inhibiting UM *in vitro* models. However, in the context of large primary tumors, intravitreal application has displayed paradoxical tumor growth [177]. Aflibercept, an additional anti-VEGF drug, has demonstrated potential efficacy in treating inoperable metastatic melanoma [178]. Research indicates that achieving the necessary antiangiogenic effect may require a combination of anti-VEGF therapy and the blockade of angiopoietin protein-like-4 (ANGPTL4) [179].

### Targeted molecular inhibitors for conjunctival melanoma

Targeted therapy or small molecule inhibitors encompass drugs designed to target genetic mutations and pathways upregulated in malignant diseases and absent in healthy tissues. This type of therapy for CM has been proposed for application in CoM and since 2013, targeted therapies have been documented in a limited number of CoM cases. According to available data, there are no ongoing clinical trials formally investigating these drugs in the context of CoM therapy. Currently, insights into treatment outcomes are drawn from small series, individual case reports, and preliminary *in vitro* studies. The main objective of systemic targeted therapy in CoM is to manage widespread local disease, particularly when it is too extensive for excision or as an alternative to orbital exenteration. Furthermore, these therapies are designed to effectively target regional and distant metastases, contributing to a comprehensive treatment approach [48,127].

Table 4 represents mutational load, mutation location, targeting driver mutations, and agents in conjunctival melanoma. The first agents introduced for CM therapy were Vemurafenib and Dabrafenib, targeting the active conformation of BRAF [180,181]. Another compound, Trametinib, developed and explored in melanoma therapy, addresses cells that have found ways to bypass BRAF inhibition and activate MEK, the downstream effector protein [182–184]. Trametinib, a MEK inhibitor, was designed for treating resistant BRAF skin melanomas or in combination with BRAF inhibitors in BRAF mutant melanomas [63,185]. In experimental studies, Vemurafenib and Dabrafenib were employed in CoM therapy, resulting in decreased growth and proliferation. The MEK inhibitor Trametinib also exhibited suppression of proliferation, displaying a dose-dependent cytotoxic effect. The combination of these two inhibitors demonstrated a synergistic effect [186].

In the literature, there are documented cases of treating CoM using BRAF inhibitor monotherapy or a combination of BRAF and MEK inhibitors. Additionally, a reported case involved the use of the anti-PD1 agent Pembrolizumab, while another case utilized the anti-PD1 agent Nivolumab in conjunction with Dabrafenib and Trametinib [187]. Combined treatment was indicated for cases involving locally advanced and metastatic disease. The outcomes of both monotherapy and combined therapy varied, encompassing complete remission, partial response, and initial improvement followed by disease progression [188,189].

AEs following targeted therapy are prevalent among patients with CM. The use of Vemurafenib in CM treatment demonstrated toxicity, with common AEs including cutaneous manifestations such as rash, keratoacanthoma, squamous cell carcinoma, as well as arthralgia, fatigue, photophobia, nausea, diarrhoea, alopecia, and liver abnormalities. Some toxic reactions manifest at the treatment’s initiation while others may occur within days, weeks, or months. Certain effects may persist while others regress over time. BRAF/MEK combination therapy can also induce similar toxic effects, alongside QT prolongation, decreased left ventricular ejection fraction, peripheral oedema and interstitial lung disease, and pneumonitis. Ocular side effects encompass uveitis, central serous retinopathy, retinal vein occlusion, and retinal pigment epithelial detachments. The observed toxicity led to dose modifications or even treatment discontinuation in 38% of treated patients with CM. Likewise, treating CoM with single BRAF inhibitors resulted in cutaneous side effects. In one patient, it manifested as arthralgia and diarrhea. The combined treatment with BRAF/MEK inhibitors triggered severe ADs, including nausea, vomiting, liver toxicity, and pyrexia [190,191].

### Future perspectives

Unlike most ophthalmological diseases, eye tumors present a dual threat by not only endangering vision but also compromising the integrity of the eye and more importantly life itself. Significant efforts are being directed toward enhancing diagnostic precision, implementing selective treatments with minimized side effects, and developing reliable screening tests to identify the systemic dissemination of malignant eye tumors in the early stages. In the context of uveal and conjunctival melanoma, primary goals include increasing the systemic efficacy of treatment for metastatic conditions and investigating the potential of adjuvant therapy to reduce the incidence of metastatic spread and associated mortality [24].

While both UM and CoM belong to the category of ocular melanomas, they represent different subtypes with varying genetic profiles. UM commonly exhibits mutations in GNAQ and GNA11 [51], while BRAF mutations are prevalent in CoM. Germline mutations in BAP1 have been observed in UM, whereas CoM is not associated with the BAP1 tumor predisposition syndrome [14,145]. The varied genetic backgrounds of these melanomas not only differentiate them but also lead to significant variations in OS rates. The prognosis for UM is less favourable compared to CoM, with their patterns of metastasis differing markedly. CoM often spreads to regional lymph nodes, whereas UM predominantly metastasizes to the liver. Despite these observed distinctions, the precise pathogenic mechanisms contributing to the survival disparities remain unclear. Current research has illuminated specific genetic markers contributing to the prognosis variations. Notably, BAP1 mutations have been associated with a poor prognosis in UM patients, and approximately half of them carry these mutations, further contributing to the grim prognosis. Additionally, splicing factor 3B subunit 1 (SF3B1) mutations are linked to a delayed onset of UM metastases. Understanding these genetic profiles provides a potential explanation for the diverse survival outcomes seen in patients with different subtypes of ocular melanoma [11,145].

Significantly, the prognosis for UM is influenced by molecular factors such as monosomy of chromosome 3 and BAP1 alterations, while conventional tumor staging remains more predictive for CoM [145]. Observable molecular differences exist between UM and CoM where UM demonstrates a low mutation burden with the MAPK signaling pathway being upregulated due to activating mutations in either GNAQ or GNA11 in approximately 80% of cases [10,145]. Additional associated genetic mutations in UM include those related to BAP1 and, rarely, KIT [8–10]. In contrast, the molecular pathogenesis of CoM more closely resembles that of cutaneous and mucosal melanoma, involving mutations in BRAF [10].

While exceptions exist, local tumor control of UM is generally no longer a primary concern and the challenge lies in anticipating future metastatic risks after eye treatment. In most cases, early intervention is preferred when a melanocytic lesion begins to display suspicious features, rather than waiting for such lesions to grow. Over the past two decades, there has been a rapid evolution in our understanding of the molecular behavior of UM, resulting in the development of more precise genetic prognostic tools to identify patients at high risk for metastatic spread. The current focus is primarily on developing immune modulatory therapy for established metastatic disease. Determining the optimal treatment for metastatic disease will provide effective therapy for use in the adjuvant setting, ultimately leading to improved survival [125,145].

Further, CoM, although rare, is a lethal malignancy affecting the ocular surface. Due to its location, it is often detected at a smaller size compared to UM, resulting in better survival rates. Primary treatment involves surgical excision and cryotherapy with additional adjuvant treatments in cases of incomplete excision. Evaluating regional lymphadenopathy is crucial, as lymphatic spread is relatively common, although distal metastasis without regional lymph node involvement has been noted in 25% of cases [5,69]. Further research is needed to revise the indications, advantages, and disadvantages of various screening methods, especially sentinel lymph node biopsy (SLNB). Genetic studies on the molecular basis of CoM, particularly regarding BRAF mutations are evolving and will undoubtedly influence decision-making in the management and treatment of this condition.

Regrettably, there is currently no effective treatment for metastatic ocular melanoma. While liver resection is an option in only a limited number of cases for UM, there is no validated systemic treatment in practice. Considering the genetic profile similarity between CoM and CM, especially those harbouring a BRAF mutation, targeted treatment with BRAF/MEK inhibitors, as employed in CM, could be considered. To enhance our understanding of ocular melanoma and potentially identify therapeutic targets, additional genetic and molecular testing is imperative [145].

## Conclusion

The molecular characteristics of CoM closely resemble those of CM. Advances in the treatment of CM, driven by a better understanding of genetics and molecular pathways in the pathogenesis of both CM and CoM, as well as an improved understanding of immune reactions in pathogenesis, have positively impacted therapy for both types of melanomas.

Presently, we have targeted management options such as molecular inhibitors: BRAF and MEK inhibitors as well as immunotherapy with ICIs, marking a new era in the treatment of these malignancies.

Recent insights into melanoma genetics and embiogenesis and the ability to characterize tumors at a molecular level provide an opportunity for more personalized treatment, even for tumors that are still localized. This advancement enhances our ability to predict the metastatic potential of the tumor, allowing for tailored treatments that differ from current approaches.

## Data Availability

Not applicable.

## References

[1] Mihajlovic, M., Vlajkovic, S., Jovanovic, P., Stefanovic, V. (2012). Primary mucosal melanomas: A comprehensive review. International Journal of Clinical and Experimental Pathology*,* 5 *(*8*),* 739–753; 23071856 PMC3466987

[2] Topić, B., Mašić, T., Radović, S., Lincender, I., Muhić, E. (2017). Primary oral mucosal melanomas-two case reports and comprehensive literature review. Acta Clinica Croatica*,* 56 *(*2*),* 323–330. 10.20471/acc.2017.56.02.17; 29485801

[3] Pandiani, C., Beranger, G. E., Leclerc, J., Ballotti, R., Bertolotto, C. (2017). Focus on cutaneous and uveal melanoma specificities. Genes & Development*,* 31 *(*8*),* 724–743. 28512236 10.1101/gad.296962.117 PMC5435887

[4] Brenner, M., Hearing, V. J. (2008). The protective role of melanin against UV damage in human skin. Photochemistry and Photobiology*,* 84 *(*3*),* 539–549; 18435612 10.1111/j.1751-1097.2007.00226.x PMC2671032

[5] Griewank, K. G., Westekemper, H., Murali, R., Mach, M., Schilling, B. et al. (2013). Conjunctival melanomas harbor BRAF and NRAS mutations and copy number changes similar to cutaneous and mucosal melanomas. Clinical Cancer Research*,* 19 *(*12*),* 3143–3152; 23633454 10.1158/1078-0432.CCR-13-0163

[6] Sarna, T. (1992). Properties and function of the ocular melanin--a photobiophysical view. Journal of Photochemistry and Photobiology B: Biology*,* 12 *(*3*),* 215–258; 1635010 10.1016/1011-1344(92)85027-r

[7] van der Kooij, MK., Speetjens, FM., Van der Burg, SH., Kapiteijn, E. (2019). Uveal versus cutaneous melanoma; same origin, very distinct tumor types. Cancers*,* 11 *(*6*),* 845; 31248118 10.3390/cancers11060845 PMC6627906

[8] Rodrigues, M., Koning, L., Coupland, S. E., Jochemsen, A. G., Marais, R. et al. (2019). So close, yet so far: discrepancies between uveal and other melanomas. A position paper from UM cure 2020. Cancers*,* 11 *(*7*),* 1032; 31336679 10.3390/cancers11071032 PMC6678543

[9] Kaštelan, S., Gverović, A. A., Beketić, O. L., Salopek, R. J., Kasun, B. et al. (2018). Conjunctival melanoma-epidemiological trends and features. Pathology & Oncology Research*,* 24 *(*4*),* 787–796. 29802540 10.1007/s12253-018-0419-3

[10] Pašalić, D., Nikuševa-Martić, T., Sekovanić, A., Kaštelan, S. (2023). Genetic and epigenetic features of uveal melanoma—An overview and clinical implications. International Journal of Molecular Sciences*,* 24 *(*16*),* 12807. 10.3390/ijms241612807; 37628989 PMC10454135

[11] Jovanovic, P., Mihajlovic, M., Djordjevic-Jocic, J., Vlajkovic, S., Cekic, S. et al. (2013). Ocular melanoma: An overview of the current status. International Journal of Clinical and Experimental Pathology*,* 6 *(*7*),* 1230–1244; 23826405 PMC3693189

[12] Kaštelan, S., Mrazovac, Z. D., Ivanković, M., Marković, I., Gverović, A. A. (2022). Liver metastasis in uveal melanoma-treatment options and clinical outcome. Frontiers in Bioscience*,* 27 *(*2*),* 72. 10.31083/j.fbl2702072 35227015

[13] Aronow, M. E., Topham, A. K., Singh, A. D. (2018). Uveal melanoma: 5-year update on incidence, treatment, and survival (SEER 1973–2013). Ocular Oncology and Pathology*,* 4 *(*3*),* 145–151. 10.1159/000480640; 29765944 PMC5939716

[14] Fallico, M., Raciti, G., Longo, A., Reibaldi, M., Bonfiglio, V. et al. (2021). Current molecular and clinical insights into uveal melanoma (Review). International Journal of Oncology*,* 58 *(*4*),* 10. 10.3892/ijo.2021.5190; 33649778 PMC7910016

[15] Cherkas, E., Kalafatis, N. E., Marous, M. R., Shields, C. L. (2024). Iris melanoma: Review of clinical features, risks, management, and outcomes. Clinics in Dermatology*,* 42 *(*1*),* 62–70. 10.1016/j.clindermatol.2023.10.009; 37865279

[16] Dockery, P. W., DeSimone, J. D., Liu, C. K., Achuck, K., Hamburger, J. et al. (2023). Effectiveness of treatment for iris melanoma: Surgical versus radiotherapeutic approaches. Canadian Journal of Ophthalmology (In Press). 10.1016/j.jcjo.2023.10.021; 38040029

[17] Melendez-Moreno, A., Yeşiltaş, Y. S., Wrenn, J., Singh, A. D. (2023). Iris melanoma: Prognostication for metastasis. Survey of Ophthalmology*,* 68 *(*5*),* 957–963. 10.1016/j.survophthal.2023.05.006; 37207703

[18] Shields, C. L., Kaliki, S., Shah, S. U., Luo, W., Furuta, M. et al. (2012). Iris melanoma: Features and prognosis in 317 children and adults. Journal of American Association for Pediatric Ophthalmology and Strabismus*,* 16 *(*1*),* 10–16; 22370659 10.1016/j.jaapos.2011.10.012

[19] Demirci, H., Shields, C. L., Shields, J. A., Eagle Jr, R. C., Honavar, S. (2001). Ring melanoma of the anterior chamber angle: A report of fourteen cases. American Journal of Ophthalmology*,* 132 *(*3*),* 336–3342; 11530045 10.1016/s0002-9394(01)01051-0

[20] Kaliki, S., Shields, C. L. (2017). Uveal melanoma: Relatively rare but deadly cancer. Eye*,* 31 *(*2*),* 241–257; 27911450 10.1038/eye.2016.275 PMC5306463

[21] Shields, C. L., Furuta, M., Thangappan, A., Nagori, S., Mashayekhi, A. et al. (2009). Metastasis of uveal melanoma millimeter-by-millimeter in 8033 consecutive eyes. Archives of Ophthalmology*,* 127 *(*8*),* 989–998; 19667335 10.1001/archophthalmol.2009.208

[22] Tarlan, B., Kıratlı, H. (2016). Uveal melanoma: Current trends in diagnosis and management. Turkish Journal of Ophthalmology*,* 46*,* 123–137; 27800275 10.4274/tjo.37431 PMC5076295

[23] Huang, Y. Y., Hou, T. Y., Yu, W. K., Tsai, C. C., Kao, S. C. et al. (2021). The clinical feature and treatment outcome of ocular melanoma: A 34-year experience in a tertiary referral center. Cancers*,* 13*,* 5926; 34885033 10.3390/cancers13235926 PMC8657155

[24] Carvajal, R. D., Sacco, J. J., Jager, M. J., Eschelman, D. J., Olofsson Bagge, R. et al. (2023). Advances in the clinical management of uveal melanoma. Nature Reviews Clinical Oncology*,* 20 *(*2*),* 99–115. 10.1038/s41571-022-00714-1; 36600005

[25] Brănişteanu, DE., Porumb-Andrese, E., Porumb, V., Stărică, A., Moraru, AD. et al. (2023). New treatment horizons in uveal and cutaneous melanoma. Life*,* 13 *(*8*),* 1666. 10.3390/life13081666; 37629523 PMC10455832

[26] Damato, B. (2018). Ocular treatment of choroidal melanoma in relation to the prevention of metastatic death—A personal view. Progress in Retinal and Eye Research*,* 66*,* 187–199. 10.1016/j.preteyeres.2018.03.004; 29571968

[27] Li, Y., Shi, J., Yang, J., Ge, S., Zhang, J. et al. (2020). Uveal melanoma: Progress in molecular biology and therapeutics. Therapeutic Advances in Medical Oncology*,* 12. 10.1177/1758835920965852 PMC7586035 33149769

[28] Shields, C. L., Shields, J. A. (2019). Tumors of the conjunctiva and cornea. Indian Journal of Ophthalmology*,* 67 *(*12*),* 1930–1948. 10.4103/ijo.IJO_2040_19; 31755426 PMC6896532

[29] Vora, G. K., Demirci, H., Marr, B., Mruthyunjaya, P. (2017). Advances in the management of conjunctival melanoma. Survey of Ophthalmology*,* 62 *(*1*),* 26–42; 27321895 10.1016/j.survophthal.2016.06.001 PMC5353981

[30] Zeiger, J. S., Lally, S. E., Dalvin, L. A., Shields, C. L. (2023). Advances in conjunctival melanoma: Clinical features, diagnostic modalities, staging, genetic markers, and management. Canadian Journal of Ophthalmology (In Press). 10.1016/j.jcjo.2023.02.003; 36921624 PMC13264506

[31] Isager, P., Engholm, G., Overgaard, J., Storm, H. (2006). Uveal and conjunctival malignant melanoma in Denmark 1943–97: Observed and relative survival of patients followed through 2002. Ophthalmic Epidemiology*,* 13 *(*2*),* 85–96; 16581612 10.1080/09286580600553330

[32] Chang, A. E., Karnell, L. H., Menck, H. R. (1998). The national cancer data base report on cutaneous and noncutaneous melanoma: A summary of 84,836 cases from the past decade. The American college of surgeons commission on cancer and the american cancer society. Cancer*,* 83 *(*8*),* 1664–1678; 9781962 10.1002/(sici)1097-0142(19981015)83:8<1664::aid-cncr23>3.0.co;2-g

[33] Wu, M., Yavuzyigitoglu, S., Brosens, E., Ramdas, W. D., Kiliç, E. (2023). Worldwide incidence of ocular melanoma and correlation with pigmentation-related risk factors. Investigative Ophthalmology & Visual Science*,* 64 *(*13*),* 45. 10.1167/iovs.64.13.45; 37902747 PMC10617638

[34] Yu, G. P., Hu, D. N., McCormick, S., Finger, P. T. (2003). Conjunctival melanoma: Is it increasing in the United States? American Journal of Ophthalmology*,* 135 *(*6*),* 800–806; 12788119 10.1016/s0002-9394(02)02288-2

[35] Mikkelsen, L. H., Andersen, M. K., Andreasen, S., Larsen, A. C., Tan, Q. et al. (2019). Global microRNA profiling of metastatic conjunctival melanoma. Melanoma Research*,* 29 *(*5*),* 465–473. 10.1097/CMR.0000000000000606; 30932942

[36] Triay, E., Bergman, L., Nilsson, B., All-Ericsson, C., Seregard, S. (2009). Time trends in the incidence of conjunctival melanoma in Sweden. British Journal of Ophthalmology*,* 93 *(*11*),* 1524–1528; 19628487 10.1136/bjo.2009.157933

[37] Zembowicz, A., Mandal, R. V., Choopong, P. (2010). Melanocytic lesions of the conjunctiva. Archives of Pathology & Laboratory Medicine*,* 134 *(*12*),* 1785–1792. 21128776 10.5858/2009-0522-RAR.1

[38] Taban, M., Traboulsi, E. I. (2007). Malignant melanoma of the conjunctiva in children: A review of the international literature 1965–2006. Journal of Pediatric Ophthalmology and Strabismus*,* 44 *(*5*),* 277–282; 17913169 10.3928/01913913-20070901-01

[39] Shields, J. A., Shields, C. L., Mashayekhi, A., Marr, B. P., Benavides, R. et al. (2007). Primary acquired melanosis of the conjunctiva: Experience with 311 eyes. Transactions of the American Ophthalmological Society*,* 105*,* 61–71; 18427595 PMC2258121

[40] Gear, H., Williams, H., Kemp, E. G., Roberts, F. (2004). BRAF mutations in conjunctival melanoma. Investigative Ophthalmology & Visual Science*,* 45 *(*8*),* 2484–2488. 15277467 10.1167/iovs.04-0093

[41] Lake, S. L., Jmor, F., Dopierala, J., Taktak, A. F., Coupland, S. E. et al. (2011). Multiplex ligation-dependent probe amplification of conjunctival melanoma reveals common BRAF V600E gene mutation and gene copy number changes. Investigative Ophthalmology & Visual Science*,* 52 *(*8*),* 5598–5604. 21693616 10.1167/iovs.10-6934

[42] Anastassiou, G., Heiligenhaus, A., Bechrakis, N., Bader, E., Bornfeld, N. et al. (2002). Prognostic value of clinical and histopathological parameters in conjunctival melanomas: A retrospective study. British Journal of Ophthalmology*,* 86 *(*2*),* 163–167; 11815341 10.1136/bjo.86.2.163 PMC1771018

[43] Shields, C. L., Silva, A. M. V., Laiton, A., Kalafatis, N. E., Schiller, E. et al. (2024). Conjunctival melanoma: Insights into classification, outcomes, and biomarkers. Clinics in Dermatology*,* 42 *(*1*),* 46–55. 10.1016/j.clindermatol.2023.10.010; 37858779

[44] Cohen, V. M. L., O’Day, R. F. (2019). Management issues in conjunctival tumours: Conjunctival melanoma and primary acquired melanosis. Ophthalmology and Therapy*,* 8 *(*4*),* 501–510. 10.1007/s40123-019-00219-8; 31691901 PMC6858423

[45] Williams Jr, B. K., di Nicola, M. (2021). Ocular oncology-primary and metastatic malignancies. Medical Clinics North America*,* 105 *(*3*),* 531–550. 10.1016/j.mcna.2021.02.005; 33926645

[46] Shields, C. L., Shields, J. A., Gunduz, K., Cater, J., Mercado, G. V. et al. (2000). Conjunctival melanoma: Risk factors for recurrence, exenteration, metastasis, and death in 150 consecutive patients. Archives of Ophthalmology*,* 118 *(*11*),* 1497–1507; 11074806 10.1001/archopht.118.11.1497

[47] Lim, L. A., Madigan, M. C., Conway, R. M. (2013). Conjunctival melanoma: A review of conceptual and treatment advances. Clinical Ophthalmology*,* 6*,* 521–531. 10.2147/OPTH.S38415; 23515569 PMC3601642

[48] Bastian, B. C. (2014). The molecular pathology of melanoma: An integrated taxonomy of melanocytic neoplasia. Annual Review of Pathology Mechanisms of Disease*,* 9 *(*1*),* 239–271. 10.1146/annurev-pathol-012513-104658; 24460190 PMC4831647

[49] Brouwer, N. J., Verdijk, R. M., Heegaard, S., Marinkovic, M., Esmaeli, B. et al. (2022). Conjunctival melanoma: New insights in tumor genetics and immunology, leading to new therapeutic options. Progress in Retinal and Eye Research*,* 86*,* 100971. 10.1016/j.preteyeres.2021.100971; 34015548

[50] Van Raamsdonk, CD., Griewank, KG., Crosby, MB., Garrido, MC., Vemula, S. et al. (2010). Mutations in GNA11 in uveal melanoma. The New-England Medical Review and Journal*,* 363 *(*23*),* 2191–2199. 10.1056/NEJMoa1000584; 21083380 PMC3107972

[51] Shoushtari, A. N., Carvajal, R. D. (2014). GNAQ and GNA11 mutations in uveal melanoma. Melanoma Research*,* 24 *(*6*),* 525–534. 10.1097/CMR.0000000000000121; 25304237

[52] Johansson, P., Aoude, L. G., Wadt, K., Glasson, W. J., Warrier, S. K. et al. (2016). Deep sequencing of uveal melanoma identifies a recurrent mutation in PLCB4. Oncotarget*,* 7 *(*4*),* 4624–4631. 10.18632/oncotarget.6614; 26683228 PMC4826231

[53] Populo, H., Vinagre, J., Lopes, J. M., Soares, P. (2011). Analysis of GNAQ mutations, proliferation and MAPK pathway activation in uveal melanomas. British Journal of Ophthalmology*,* 95 *(*5*),* 715–719. 10.1136/bjo.2009.174417; 20805136

[54] Steeb, T., Wessely, A., Ruzicka, T., Heppt, M. V., Berking, C. (2018). How to MEK the best of uveal melanoma: A systematic review on the efficacy and safety of MEK inhibitors in metastatic or unresectable uveal melanoma. European Journal of Cancer*,* 103*,* 41–51. 10.1016/j.ejca.2018.08.005; 30205280

[55] Carnero, A., Paramio, J. M. (2014). The PTEN/PI3K/AKT pathway *in vivo*, cancer mouse models. Frontiers in Oncology*,* 4*,* 252. 10.3389/fonc.2014.00252; 25295225 PMC4172058

[56] Feng, X., Degese, M. S., Iglesias-Bartolome, R., Vaque, J. P., Molinolo, A. A. et al. (2014). Hippo-independent activation of YAP by the GNAQ uveal melanoma oncogene through a trio-regulated rho GTPase signaling circuitry. Cancer Cell*,* 25 *(*6*),* 831–845. 10.1016/j.ccr.2014.04.016; 24882515 PMC4074519

[57] Onken, M. D., Worley, L. A., Ehlers, J. P., Harbour, J. W. (2004). Gene expression profiling in uveal melanoma reveals two molecular classes and predicts metastatic death. Cancer Research*,* 64 *(*20*),* 7205–7209. 10.1158/0008-5472.CAN-04-1750; 15492234 PMC5407684

[58] Beek JGMv, Koopmans, AE., Verdijk, RM., Naus, NC., Klein et al. (2013). Diagnosis, histopathologic and genetic classification of uveal melanoma. Rijeka: IntechOpenv.

[59] Tschentscher, F., Prescher, G., Horsman, D. E., White, V. A., Rieder, H. et al. (2001). Partial deletions of the long and short arm of chromosome 3 point to two tumor suppressor genes in uveal melanoma. Cancer Research*,* 61 *(*8*),* 3439–3442; 11309305

[60] Scholes, A. G., Damato, B. E., Nunn, J., Hiscott, P., Grierson, I. et al. (2003). Monosomy 3 in uveal melanoma: Correlation with clinical and histologic predictors of survival. Investigative Opthalmology & Visual Science*,* 44 *(*3*),* 1008–1011. 10.1167/iovs.02-0159; 12601021

[61] Ehlers, J. P., Worley, L., Onken, M. D., Harbour, J. W. (2008). Integrative genomic analysis of aneuploidy in uveal melanoma. Clinical Cancer Research*,* 14 *(*1*),* 115–122. 10.1158/1078-0432.CCR-07-1825; 18172260

[62] Kilic, E., Van Gils, W., Lodder, E., Beverloo, H. B., Van Til, M. E. et al. (2006). Clinical and cytogenetic analyses in uveal melanoma. Investigative Ophthalmology & Visual Science*,* 47 *(*9*),* 3703–3707. 10.1167/iovs.06-0101; 16936076

[63] Cao, J., Heijkants, R. C., Jochemsen, A. G., Dogrusoz, M., de Lange, M. J. et al. (2017). Targeting of the MAPK and AKT pathways in conjunctival melanoma shows potential synergy. Oncotarget*,* 8 *(*35*),* 58021–58036. 10.18632/oncotarget.10770; 28938534 PMC5601630

[64] Kenawy, N., Kalirai, H., Sacco, J. J., Lake, S. L., Heegaard, S. et al. (2019). Conjunctival melanoma copy number alterations and correlation with mutation status, tumor features, and clinical outcome. Pigment Cell & Melanoma Research*,* 32 *(*4*),* 564–575. 10.1111/pcmr.12767; 30672666 PMC6849808

[65] Bol, K. F., Donia, M., Heegaard, S., Kiilgaard, J. F., Svane, I. M. (2020). Genetic biomarkers in melanoma of the ocular region: What the medical oncologist should know. International Journal of Molecular Sciences*,* 21 *(*15*),* 5231. 10.3390/ijms21155231; 32718045 PMC7432371

[66] Jakob, J. A., Bassett Jr, R. L., Ng, C. S., Curry, J. L., Joseph, R. W. et al. (2012). NRAS mutation status is an independent prognostic factor in metastatic melanoma. Cancer*,* 118 *(*16*),* 4014–4023. 10.1002/cncr.26724; 22180178 PMC3310961

[67] Cichowski, K., Jacks, T. (2001). NF1 tumor suppressor gene function: Narrowing the GAP. Cell*,* 104 *(*4*),* 593–604. 10.1016/S0092-8674(01)00245-8; 11239415

[68] Populo, H., Soares, P., Rocha, A. S., Silva, P., Lopes, J. M. (2010). Evaluation of the mTOR pathway in ocular (uvea and conjunctiva) melanoma. Melanoma Research*,* 20 *(*2*),* 107–117. 10.1097/CMR.0b013e32832ccd09; 20173664

[69] Swaminathan, S. S., Field, M. G., Sant, D., Wang, G., Galor, A. et al. (2017). Molecular characteristics of conjunctival melanoma using whole-exome sequencing. JAMA Ophthalmology*,* 135 *(*12*),* 1434–1437. 10.1001/jamaophthalmol.2017.4837; 29121185 PMC5782797

[70] Griewank, K. G., Murali, R., Schilling, B., Scholz, S., Sucker, A. et al. (2013). TERT promoter mutations in ocular melanoma distinguish between conjunctival and uveal tumors. British Journal of Cancer*,* 109 *(*2*),* 497–501. 10.1038/bjc.2013.312; 23799844 PMC3721405

[71] Mitchell, B., Mahalingam, M. (2014). The CXCR4/CXCL12 axis in cutaneous malignancies with an emphasis on melanoma. Histology and Histopathology*,* 29 *(*12*),* 1539–1546. 10.14670/HH-29.1539; 24879309

[72] Nagarsheth, N., Wicha, M. S., Zou, W. (2017). Chemokines in the cancer microenvironment and their relevance in cancer immunotherapy. Nature Reviews Immunology*,* 17 *(*9*),* 559–572. 10.1038/nri.2017.49; 28555670 PMC5731833

[73] Mishan, M. A., Ahmadiankia, N., Bahrami, A. R. (2016). CXCR4 and CCR7: Two eligible targets in targeted cancer therapy. Cell Biology International*,* 40 *(*9*),* 955–967. 10.1002/cbin.10631; 27248053

[74] Van Ipenburg, J. A., de Waard, N. E., Naus, N. C., Jager, M. J., Paridaens, D. et al. (2019). Chemokine receptor expression pattern correlates to progression of conjunctival melanocytic lesions. Investigative Ophthalmology & Visual Science*,* 60 *(*8*),* 2950–2957. 10.1167/iovs.19-27162; 31305861

[75] Larsen, A. C., Mikkelsen, L. H., Borup, R., Kiss, K., Toft, P. B. et al. (2016). MicroRNA expression profile in conjunctival melanoma. Investigative Ophthalmology and Visual Science*,* 57 *(*10*),* 4205–4212. 10.1167/iovs.16-19862; 27548891

[76] Worley, L. A., Long, M. D., Onken, M. D., Harbour, J. W. (2008). Micro-RNAs associated with metastasis in uveal melanoma identified by multiplexed microarray profiling. Melanoma Research*,* 18 *(*3*),* 184–190. 10.1097/CMR.0b013e3282feeac6; 18477892

[77] Stark, M. S., Gray, E. S., Isaacs, T., Chen, F. K., Millward, M. et al. (2019). A panel of circulating microRNAs detects uveal melanoma with high precision. Translational Vision Science and Technology*,* 18 *(*6*),* 12. 10.1167/tvst.8.6.12; 31737436 PMC6855372

[78] Iacona, J. R., Lutz, CS. (2019). miR-146a-5p: Expression, regulation, and functions in cancer. Wiley Interdisciplinary Reviews: RNA*,* 10 *(*4*),* e1533. 10.1002/wrna.1533; 30895717

[79] Russo, A., Caltabiano, R., Longo, A., Avitabile, T., Franco, L. et al. (2016). Increased levels of miRNA-146a in serum and histologic samples of patients with uveal melanoma. Frontiers in Pharmacology*,* 7*,* 424. 10.3389/fphar.2016.00424; 27895580 PMC5108814

[80] Venkatesan, N., Kanwar, J., Deepa, P. R., Khetan, V., Crowley, T. M. et al. (2016). Clinico-pathological association of delineated miRNAs in uveal melanoma with monosomy 3/disomy 3 chromosomal aberrations. PLoS One*,* 11 *(*1*),* e0146128. 10.1371/journal.pone.0146128; 26812476 PMC4728065

[81] Sand, M., Skrygan, M., Sand, D., Georgas, D., Gambichler, T. et al. (2013). Comparative microarray analysis of microRNA expression profiles in primary cutaneous malignant melanoma, cutaneous malignant melanoma metastases, and benign melanocytic nevi. Cell and Tissue Research*,* 351 *(*1*),* 85–98. 10.1007/s00441-012-1514-5; 23111773

[82] Smit, K. N., Chang, J., Derks, K., Vaarwater, J., Brands, T. et al. (2019). Aberrant microRNA expression and its implications for uveal melanoma metastasis. Cancers*,* 11 *(*6*),* 815. 10.3390/cancers11060815; 31212861 PMC6628189

[83] Broseghini, E., Dika, E., Londin, E., Ferracin, M. (2021). MicroRNA isoforms contribution to melanoma pathogenesis. Non-Coding RNA*,* 7 *(*4*),* 63. 10.3390/ncrna7040063; 34698264 PMC8544706

[84] DiVincenzo, M. J., Schwarz, E., Ren, C., Barricklow, Z., Moufawad, M. et al. (2023). Expression patterns of microRNAs and associated target genes in ulcerated primary cutaneous melanoma. Journal of Investigative Dermatology*,* 143 *(*4*),* 630–638.e3. 10.1016/j.jid.2022.09.654; 36202232

[85] Falzone, L., Romano, G. L., Salemi, R., Bucolo, C., Tomasello, B. et al. (2019). Prognostic significance of deregulated microRNAs in uveal melanomas. Molecular Medicine Reports*,* 19 *(*4*),* 2599–2610. 10.3892/mmr.2019.9949; 30816460 PMC6423615

[86] Triozzi, P. L., Achberger, S., Aldrich, W., Crabb, J. W., Saunthararajah, Y. et al. (2016). Association of tumor and plasma microRNA expression with tumor monosomy-3 in patients with uveal melanoma. Clinical Epigenetics*,* 8*,* 80. 10.1186/s13148-016-0243-0; 27453764 PMC4957327

[87] Vashishtha, A., Lee, T. J., Sharma, A., Wallbillich, J. J. (2020). Research paper changes in microRNA expression associated with metastasis and survival in patients with uveal melanoma. Oncotarget*,* 11 *(*16*),* 1435–1447. 10.18632/oncotarget.27559; 32363001 PMC7185062

[88] Wróblewska, J. P., Lach, M. S., Ustaszewski, A., Kulcenty, K., Ibbs, M. et al. (2020). The potential role of selected miRNA in uveal melanoma primary tumors as early biomarkers of disease progression. Genes*,* 11 *(*3*),* 271. 10.3390/genes11030271; 32131485 PMC7140886

[89] Li, Y., Li, M., Shats, I., Krahn, J. M., Flake, G. P. et al. (2019). Glypican 6 is a putative biomarker for metastatic progression of cutaneous melanoma. PLoS One*,* 14 *(*6*),* e0218067. 10.1371/journal.pone.0218067; 31199813 PMC6568403

[90] Mueller, D. W., Rehli, M., Bosserhoff, A. K. (2009). MiRNA expression profiling in melanocytes and melanoma cell lines reveals miRNAs associated with formation and progression of malignant melanoma. Journal of Investigative Dermatology*,* 29 *(*7*),* 1740–1751. 10.1038/jid.2008.452; 19212343

[91] Mumford, S. L., Towler, B. P., Pashler, A. L., Gilleard, O., Martin, Y. et al. (2018). Circulating microRNA biomarkers in melanoma: Tools and challenges in personalised medicine. Biomolecules*,* 8 *(*2*),* 21. 10.3390/biom8020021; 29701682 PMC6022922

[92] Jurmeister, P., Wrede, N., Hoffmann, I., Vollbrecht, C., Heim, D. et al. (2022). Mucosal melanomas of different anatomic sites share a common global DNA methylation profile with cutaneous melanoma but show location-dependent patterns of genetic and epigenetic alterations. Journal of Pathology*,* 256 *(*1*),* 61–70. 10.1002/path.5808; 34564861

[93] Yang, L., Wang, G., Shi, H., Jia, S., Ruan, J. et al. (2022). Methylation-driven gene DLL3 is a potential prognostic biomarker in ocular melanoma correlating with metastasis. Frontiers in Oncology*,* 12*,* 964902. 10.3389/fonc.2022.964902; 36338696 PMC9630341

[94] Smit, K. N., Boers, R., Vaarwater, J., Boers, J., Brands, T. et al. (2022). Genome-wide aberrant methylation in primary metastatic UM and their matched metastases. Scientific Report*,* 12 *(*1*),* 42. 10.1038/s41598-021-03964-8; 34997020 PMC8742000

[95] Li, B., Carey, M., Workman, J. L. (2007). The role of chromatin during transcription. Cell*,* 128*,* 707–719; 17320508 10.1016/j.cell.2007.01.015

[96] Holling, T. M., Bergevoet, M. W., Wilson, L., Van Eggermond, M. C., Schooten, E. et al. (2007). A role for EZH2 in silencing of IFN-γ inducible *MHC2TA* transcription in uveal melanoma. Journal of Immunology*,* 179*,* 5317–5325. 10.4049/jimmunol.179.8.5317 17911618

[97] Ding, X., Wang, X., Lin, M., Xing, Y., Ge, S. et al. (2016). *PAUPAR* lncRNA suppresses tumorigenesis by H3K4 demethylation in uveal melanoma. FEBS Letters*,* 590*,* 1729–1738; 27214741 10.1002/1873-3468.12220

[98] Landreville, S., Agapova, O. A., Matatall, K. A., Kneass, Z. T., Onken, M. D. et al. (2012). Histone deacetylase inhibitors induce growth arrest and differentiation in uveal melanoma. Clinical Cancer Research*,* 18*,* 408–416; 22038994 10.1158/1078-0432.CCR-11-0946 PMC3261307

[99] Wierenga, A. P. A., Cao, J., Luyten, G. P. M., Jager, M. J. (2019). Immune checkpoint inhibitors in uveal and conjunctival melanoma. International Ophthalmology Clinics*,* 59 *(*2*),* 53–63; 30908279 10.1097/IIO.0000000000000263

[100] Fagone, P., Caltabiano, R., Russo, A., Lupo, G., Anfuso, C. D. et al. (2017). Identification of novel chemotherapeutic strategies for metastatic uveal melanoma. Scientific Reports*,* 7*,* 44564; 28303962 10.1038/srep44564 PMC5355998

[101] Jager, M. J., Dogrusoz, M., Woodman, S. E. (2017). Uveal melanoma: Identifying immunological and chemotherapeutic targets to treat metastases. Asia-Pacific Journal of Ophthalmology*,* 6 *(*2*),* 179–185; 28399339 10.22608/APO.201782

[102] Oliva, M., Rullan, A. J., Piulats, J. M. (2016). Uveal melanoma as a target for immune-therapy. Annals of Translational Medicine*,* 4 *(*9*),* 172; 27275485 10.21037/atm.2016.05.04 PMC4876283

[103] Breazzano, M. P., Milam, R. W., Jr., Batson, S. A., Johnson, D. B., Daniels, A. B. (2017). Immunotherapy for uveal melanoma. International Ophthalmology Clinics*,* 57 *(*1*),* 29–39; 27898611 10.1097/IIO.0000000000000148

[104] Yang, W., Chen, P. W., Li, H., Alizadeh, H., Niederkorn, J. Y. (2008). PD-L1: PD-1 interaction contributes to the functional suppression of T-cell responses to human uveal melanoma cells *in vitro*. Investigative Ophthalmology & Visual Science*,* 49 *(*6*),* 2518–2525. 18296654 10.1167/iovs.07-1606 PMC2465808

[105] Apte, R. S., Mayhew, E., Niederkorn, J. Y. (1997). Local inhibition of natural killer cell activity promotes the progressive growth of intraocular tumors. Investigative Ophthalmology & Visual Science*,* 38 *(*6*),* 1277–1282. 9152248

[106] Heppt, M. V., Steeb, T., Schlager, J. G., Rosumeck, S., Dressler, C. et al. (2017). Immune checkpoint blockade for unresectable or metastatic uveal melanoma: A systematic review. Cancer Treatment Reviews*,* 60*,* 44–52; 28881222 10.1016/j.ctrv.2017.08.009

[107] Dunn, G. P., Bruce, A. T., Ikeda, H., Old, L. J., Schreiber, R. D. (2002). Cancer immunoediting: From immunosurveillance to tumor escape. Nature Immunology*,* 3 *(*11*),* 991–998; 12407406 10.1038/ni1102-991

[108] Mamalis, A., Garcha, M., Jagdeo, J. (2014). Targeting the PD-1 pathway: A promising future for the treatment of melanoma. Archives of Dermatological Research*,* 306 *(*6*),* 511–519; 24615548 10.1007/s00403-014-1457-7 PMC4110159

[109] Field, M. G., Decatur, C. L., Kurtenbach, S., Gezgin, G., Van der Velden, P. A., et al. (2016). PRAME as an independent biomarker for metastasis in uveal melanoma. Clinical Cancer Research*,* 22 *(*5*),* 1234–1242; 26933176 10.1158/1078-0432.CCR-15-2071 PMC4780366

[110] Teng, M. W., Ngiow, S. F., Ribas, A., Smyth, M. J. (2015). Classifying cancers based on T-cell infiltration and PD-L1. Cancer Research*,* 75 *(*11*),* 2139–2145; 25977340 10.1158/0008-5472.CAN-15-0255 PMC4452411

[111] Alvarez-Rodriguez, B., Latorre, A., Posch, C., Somoza, A. (2017). Recent advances in uveal melanoma treatment. Medicinal Research Reviews*,* 37 *(*6*),* 1350–1372; 28759124 10.1002/med.21460

[112] Niederkorn, J. Y. (2009). Immune escape mechanisms of intraocular tumors. Progress in Retinal and Eye Research*,* 28 *(*5*),* 329–347; 19563908 10.1016/j.preteyeres.2009.06.002 PMC2727063

[113] Zimmer, L., Vaubel, J., Mohr, P., Hauschild, A., Utikal, J. et al. (2015). Phase II DeCOG-study of ipilimumab in pretreated and treatment-naive patients with metastatic uveal melanoma. PLoS One*,* 10 *(*3*),* e0118564; 25761109 10.1371/journal.pone.0118564 PMC4356548

[114] Maio, M., Danielli, R., Chiarion-Sileni, V., Pigozzo, J., Parmiani, G. et al. (2013). Efficacy and safety of ipilimumab in patients with pre-treated, uveal melanoma. Annals of Oncology*,* 24 *(*11*),* 2911–2915; 24067719 10.1093/annonc/mdt376

[115] Kelderman, S., Van der Kooij, M. K., Van den Eertwegh, A. J., Soetekouw, P. M., Jansen, R. L. et al. (2013). Ipilimumab in pretreated metastatic uveal melanoma patients. Results of the dutch working group on immunotherapy of oncology (WIN-O). Acta Oncologica*,* 52 *(*8*),* 1786–1788; 23607756 10.3109/0284186X.2013.786839

[116] Luke, J. J., Callahan, M. K., Postow, M. A., Romano, E., Ramaiya, N. et al. (2013). Clinical activity of ipilimumab for metastatic uveal melanoma: A retrospective review of the Dana-Farber Cancer Institute, Massachusetts General Hospital, Memorial Sloan-Kettering Cancer Center, and University Hospital of Lausanne experience. Cancer*,* 119 *(*20*),* 3687–3695; 23913718 10.1002/cncr.28282 PMC3986037

[117] Kottschade, L. A., McWilliams, R. R., Markovic, S. N., Block, M. S., Villasboas Bisneto, J. et al. (2016). The use of pembrolizumab for the treatment of metastatic uveal melanoma. Melanoma Research*,* 26 *(*3*),* 300–303; 26848796 10.1097/CMR.0000000000000242

[118] Algazi, A. P., Tsai, K. K., Shoushtari, A. N., Munhoz, R. R., Eroglu, Z. et al. (2016). Clinical outcomes in metastatic uveal melanoma treated with PD-1 and PD-L1 antibodies. Cancer*,* 122 *(*21*),* 3344–3353; 27533448 10.1002/cncr.30258 PMC5767160

[119] Bender, C., Enk, A., Gutzmer, R., Hassel, J. C. (2017). Anti-PD-1 antibodies in metastatic uveal melanoma: A treatment option? Cancer Medicine*,* 6 *(*7*),* 1581–1586; 28639409 10.1002/cam4.887 PMC5504332

[120] Schadendorf, D., Ascierto, P. A., Haanen, J. B. A. G., Espinosa, E., Demidov, L. V. et al. (2017). Efficacy and safety of nivolumab (NIVO) in patients with advanced melanoma (MEL) and poor prognostic factors who progressed on or after ipilimumab (IPI): Results from a phase II study (CheckMate 172). Journal of Clinical Oncology*,* 35 *(*15*),* 9524.

[121] Heppt, M. V., Heinzerling, L., Kähler, K. C., Forschner, A., Kirchberger, M. C. et al. (2017). Prognostic factors and outcomes in metastatic uveal melanoma treated with programmed cell death-1 or combined PD-1/cytotoxic T-lymphocyte antigen-4 inhibition. European Journal of Cancer*,* 82*,* 56–65; 28648699 10.1016/j.ejca.2017.05.038

[122] Buder-Bakhaya, K., Hassel, J. C. (2018). Biomarkers for clinical benefit of immune checkpoint inhibitor treatment—A review from the melanoma perspective and beyond. Frontiers in Immunology*,* 9*,* 1474; 30002656 10.3389/fimmu.2018.01474 PMC6031714

[123] Javed, A., Arguello, D., Johnston, C., Gatalica, Z., Terai, M. et al. (2017). PD-L1 expression in tumor metastasis is different between uveal melanoma and cutaneous melanoma. Immunotherapy*,* 9 *(*16*),* 1323–1330; 29185395 10.2217/imt-2017-0066

[124] Danilova, L., Wang, H., Sunshine, J., Kaunitz, G. J., Cottrell, T. R. et al. (2016). Association of PD-1/PD-L axis expression with cytolytic activity, mutational load, and prognosis in melanoma and other solid tumors. Proceedings of the National Academy of Sciences of the United States of America*,* 113 *(*48*),* E7769–E7777; 27837027 10.1073/pnas.1607836113 PMC5137776

[125] Kaštelan, S., Antunica Gverović, A., Oresković Beketić, L., Pelčić, G., Kasum, E. et al. (2020). Immunotherapy for Uveal Melanoma-current knowledge and perspectives. Current Medicinal Chemistry*,* 27 *(*8*),* 1350–1366; 31272342 10.2174/0929867326666190704141444

[126] Jindal, V. (2018). Role of immune checkpoint inhibitors and novel immunotherapies in uveal melanoma. Chinese Clinical Oncology*,* 7 *(*1*),* 8; 29486567 10.21037/cco.2018.01.05

[127] Kreidieh, F. Y., Tawbi, H. A. (2023). The introduction of LAG-3 checkpoint blockade in melanoma: Immunotherapy landscape beyond PD-1 and CTLA-4 inhibition. Therapeutic Advances in Medical Oncology*,* 15*,* 17588359231186027; 37484526 10.1177/17588359231186027 PMC10357068

[128] Kashyap, S., Singh, M. K., Kumar, N., Jha, J., Lomi, N. et al. (2023). Implications of LAG3 and CTLA4 immune checkpoints beyond PD-1/PD-L1 as a potential target in determining the prognosis of uveal melanoma patients. The British Journal of Ophthalmology*,* 14*,* bjo-2022-322913. 10.1136/bjo-2022-322913 36918273

[129] Hodi, F. S., O’Day, S. J., McDermott, D. F., Weber, R. W., Sosman, J. A. et al. (2010). Improved survival with ipilimumab in patients with metastatic melanoma. The New England Journal of Medicine*,* 363 *(*8*),* 711–723; 20525992 10.1056/NEJMoa1003466 PMC3549297

[130] Finger, P. T., Pavlick, A. C. (2019). Checkpoint inhibition immunotherapy for advanced local and systemic conjunctival melanoma: A clinical case series. Journal for Immunotherapy of Cancer*,* 7 *(*1*),* 83; 30909967 10.1186/s40425-019-0555-7 PMC6434860

[131] Sagiv, O., Thakar, S. D., Kandl, T. J., Ford, J., Sniegowski, M. C. et al. (2018). Immunotherapy with programmed cell death 1 inhibitors for 5 patients with conjunctival melanoma. JAMA Ophthalmology*,* 136 *(*11*),* 1236–1241; 30352118 10.1001/jamaophthalmol.2018.3488 PMC6248169

[132] Kini, A., Fu, R., Compton, C., Miller, D. M., Ramasubramanian, A. (2017). Pembrolizumab for recurrent conjunctival melanoma. JAMA Ophthalmology*,* 135 *(*8*),* 891–892; 28715523 10.1001/jamaophthalmol.2017.2279

[133] Komatsubara, K. M., Carvajal, R. D. (2017). Immunotherapy for the treatment of uveal melanoma: Current status and emerging therapies. Current Oncology Reports*,* 19 *(*7*),* 45; 28508938 10.1007/s11912-017-0606-5

[134] Ramos-Casals, M., Brahmer, J. R., Callahan, M. K., Flores-Chavez, A., Keegan, N. et al. (2020). Immune-related adverse events of checkpoint inhibitors. Nature Reviews*,* 6 *(*1*),* 38; 32382051 10.1038/s41572-020-0160-6 PMC9728094

[135] Coureau, M., Meert, A. P., Berghmans, T., Grigoriu, B. (2020). Efficacy and toxicity of immune-checkpoint inhibitors in patients with preexisting autoimmune disorders. Frontiers in Medicine*,* 7*,* 137; 32457912 10.3389/fmed.2020.00137 PMC7220995

[136] Wang, W., Lam, W. C., Chen, L. (2019). Recurrent grade 4 panuveitis with serous retinal detachment related to nivolumab treatment in a patient with metastatic renal cell carcinoma. Cancer Immunology, Immunotherapy*,* 68 *(*1*),* 85–95; 30311026 10.1007/s00262-018-2260-7 PMC11028325

[137] Papavasileiou, E., Prasad, S., Freitag, S. K., Sobrin, L., Lobo, A. M. (2016). Ipilimumab-induced ocular and orbital inflammation--A case series and review of the literature. Ocular Immunology and Inflammation*,* 24 *(*2*),* 140–146; 25760920 10.3109/09273948.2014.1001858

[138] Friedman, C. F., Clark, V., Raikhel, A. V., Barz, T., Shoushtari, A. N. et al. (2017). Thinking critically about classifying adverse events: Incidence of pancreatitis in patients treated with Nivolumab + Ipilimumab. Journal of the National Cancer Institute*,* 109 *(*4*),* djw260; 28040701 10.1093/jnci/djw260 PMC5441295

[139] Johnson, D. B., Balko, J. M., Compton, M. L., Chalkias, S., Gorham, J. et al. (2016). Fulminant myocarditis with combination immune checkpoint blockade. The New England Journal of Medicine*,* 375 *(*18*),* 1749–1755; 27806233 10.1056/NEJMoa1609214 PMC5247797

[140] Seki, T., Yasuda, A., Oki, M., Kitajima, N., Takagi, A. et al. (2017). Secondary adrenal insufficiency following nivolumab therapy in a patient with metastatic renal cell carcinoma. The Tokai Journal of Experimental and Clinical Medicine*,* 42 *(*3*),* 115–120; 28871578

[141] Kastrisiou, M., Kostadima, F. L., Kefas, A., Zarkavelis, G., Kapodistrias, N. et al. (2017). Nivolumab-induced hypothyroidism and selective pituitary insufficiency in a patient with lung adenocarcinoma: A case report and review of the literature. ESMO Open*,* 2 *(*4*),* e000217; 29067215 10.1136/esmoopen-2017-000217 PMC5640091

[142] Jonas, R. A., Rokohl, A. C., Heindl, L. M. (2020). Targeted therapy for malignant ocular melanomas. Annals of Eye Science*,* 6*,* 7.

[143] Violanti, S. S., Bononi, I., Gallenga, C. E., Martini, F., Tognon, M. et al. (2019). New insights into molecular oncogenesis and therapy of uveal melanoma. Cancers*,* 11 *(*5*),* 694; 31109147 10.3390/cancers11050694 PMC6562554

[144] Van Raamsdonk, C. D., Bezrookove, V., Green, G., Bauer, J., Gaugler, L. et al. (2009). Frequent somatic mutations of GNAQ in uveal melanoma and blue naevi. Nature*,* 457 *(*7229*),* 599–602; 19078957 10.1038/nature07586 PMC2696133

[145] Van Poppelen, N. M., de Bruyn, D. P., Bicer, T., Verdijk, R., Naus, N. et al. (2020). Genetics of ocular melanoma: Insights into genetics, inheritance and testing. International Journal of Molecular Sciences*,* 22 *(*1*),* 336; 33396957 10.3390/ijms22010336 PMC7795687

[146] Moore, A. R., Ceraudo, E., Sher, J. J., Guan, Y., Shoushtari, A. N. et al. (2016). Recurrent activating mutations of G-protein-coupled receptor CYSLTR2 in uveal melanoma. Nature Genetics*,* 48 *(*6*),* 675–680; 27089179 10.1038/ng.3549 PMC5032652

[147] Ambrosini, G., Musi, E., Ho, A. L., de Stanchina, E., Schwartz, G. K. (2013). Inhibition of mutant GNAQ signaling in uveal melanoma induces AMPK-dependent autophagic cell death. Molecular Cancer Therapeutics*,* 12 *(*5*),* 768–776; 23443802 10.1158/1535-7163.MCT-12-1020

[148] Li, Y., He, J., Qiu, C., Shang, Q., Qian, G. et al. (2019). The oncolytic virus H101 combined with GNAQ siRNA-mediated knockdown reduces uveal melanoma cell viability. Journal of Cellular Biochemistry*,* 120 *(*4*),* 5766–5776; 30320917 10.1002/jcb.27863

[149] Posch, C., Latorre, A., Crosby, M. B., Celli, A., Latorre, A. et al. (2015). Detection of GNAQ mutations and reduction of cell viability in uveal melanoma cells with functionalized gold nanoparticles. Biomedical Microdevices*,* 17 *(*1*),* 15; 25653058 10.1007/s10544-014-9908-7 PMC4586106

[150] Maziarz, M., Leyme, A., Marivin, A., Luebbers, A., Patel, P. P. et al. (2018). Atypical activation of the G protein Gα_q_ by the oncogenic mutation Q209P. The Journal of Biological Chemistry*,* 293 *(*51*),* 19586–19599. 10.1074/jbc.RA118.005291; 30352874 PMC6314142

[151] Onken, M. D., Makepeace, C. M., Kaltenbronn, K. M., Kanai, S. M., Todd, T. D. et al. (2018). Targeting nucleotide exchange to inhibit constitutively active G protein alpha subunits in cancer cells. Science Signaling*,* 11 *(*546*),* eaao6852; 30181242 10.1126/scisignal.aao6852 PMC6279241

[152] Yoo, J. H., Shi, D. S., Grossmann, A. H., Sorensen, L. K., Tong, Z. et al. (2016). ARF6 is an actionable node that orchestrates oncogenic GNAQ signaling in uveal melanoma. Cancer Cell*,* 29 *(*6*),* 889–904; 27265506 10.1016/j.ccell.2016.04.015 PMC5027844

[153] Ambrosini, G., Pratilas, C. A., Qin, L. X., Tadi, M., Surriga, O. et al. (2012). Identification of unique MEK-dependent genes in GNAQ mutant uveal melanoma involved in cell growth, tumor cell invasion, and MEK resistance. Clinical Cancer Research*,* 18 *(*13*),* 3552–3561; 22550165 10.1158/1078-0432.CCR-11-3086 PMC3433236

[154] Von Euw, E., Atefi, M., Attar, N., Chu, C., Zachariah, S. et al. (2012). Antitumor effects of the investigational selective MEK inhibitor TAK733 against cutaneous and uveal melanoma cell lines. Molecular Cancer*,* 11*,* 22; 22515704 10.1186/1476-4598-11-22 PMC3444881

[155] Carvajal, R. D., Sosman, J. A., Quevedo, J. F., Milhem, M. M., Joshua, A. M. et al. (2014). Effect of selumetinib vs chemotherapy on progression-free survival in uveal melanoma: A randomized clinical trial. The Journal of the American Medical Association*,* 311 *(*23*),* 2397–2405; 24938562 10.1001/jama.2014.6096 PMC4249701

[156] Wu, X., Li, J., Zhu, M., Fletcher, J. A., Hodi, F. S. (2012). Protein kinase C inhibitor AEB071 targets ocular melanoma harboring GNAQ mutations via effects on the PKC/Erk1/2 and PKC/NF-κB pathways. Molecular Cancer Therapeutics*,* 11 *(*9*),* 1905–1914. 10.1158/1535-7163.MCT-12-0121; 22653968 PMC3992123

[157] Musi, E., Ambrosini, G., de Stanchina, E., Schwartz, G. K. (2014). The phosphoinositide 3-kinase α selective inhibitor BYL719 enhances the effect of the protein kinase C inhibitor AEB071 in GNAQ/GNA11-mutant uveal melanoma cells. Molecular Cancer Therapeutics*,* 13 *(*5*),* 1044–1053; 24563540 10.1158/1535-7163.MCT-13-0550 PMC4146424

[158] Shoushtari, A. N., Ong, L. T., Schoder, H., Singh-Kandah, S., Abbate, K. T. et al. (2016). A phase 2 trial of everolimus and pasireotide long-acting release in patients with metastatic uveal melanoma. Melanoma Research*,* 26 *(*3*),* 272–277; 26795274 10.1097/CMR.0000000000000234 PMC5553199

[159] Amirouchene-Angelozzi, N., Frisch-Dit-Leitz, E., Carita, G., Dahmani, A., Raymondie, C. et al. (2016). The mTOR inhibitor Everolimus synergizes with the PI3K inhibitor GDC0941 to enhance anti-tumor efficacy in uveal melanoma. Oncotarget*,* 7 *(*17*),* 23633–23646. 10.18632/oncotarget.8054; 26988753 PMC5029652

[160] Zhao, B., Ye, X., Yu, J., Li, L., Li, W. et al. (2008). TEAD mediates YAP-dependent gene induction and growth control. Genes & Development*,* 22 *(*14*),* 1962–1971. 18579750 10.1101/gad.1664408 PMC2492741

[161] Field, M. G., Harbour, J. W. (2014). GNAQ/11 mutations in uveal melanoma: Is YAP the key to targeted therapy? Cancer Cell*,* 25 *(*6*),* 714–715; 24937456 10.1016/j.ccr.2014.05.028 PMC4966161

[162] Feng, X., Arang, N., Rigiracciolo, D. C., Lee, J. S., Yeerna, H. et al. (2019). A platform of synthetic lethal gene interaction networks reveals that the GNAQ uveal melanoma oncogene controls the hippo pathway through FAK. Cancer Cell*,* 35 *(*3*),* 457-72 e5. 30773340 10.1016/j.ccell.2019.01.009 PMC6737937

[163] Bradner, J. E., Hnisz, D., Young, R. A. (2017). Transcriptional addiction in cancer. Cell*,* 168 *(*4*),* 629–643; 28187285 10.1016/j.cell.2016.12.013 PMC5308559

[164] Falkenberg, K. J., Johnstone, R. W. (2014). Histone deacetylases and their inhibitors in cancer, neurological diseases and immune disorders. Nature Reviews Drug Discovery*,* 13 *(*9*),* 673–691. 10.1038/nrd4360; 25131830

[165] Moschos, M. M., Dettoraki, M., Androudi, S., Kalogeropoulos, D., Lavaris, A. et al. (2018). The role of histone deacetylase inhibitors in uveal melanoma: Current evidence. Anticancer Research*,* 38 *(*7*),* 3817–3824; 29970501 10.21873/anticanres.12665

[166] Amaro, A., Mirisola, V., Angelini, G., Musso, A., Tosetti, F. et al. (2013). Evidence of epidermal growth factor receptor expression in uveal melanoma: Inhibition of epidermal growth factor-mediated signalling by Gefitinib and Cetuximab triggered antibody-dependent cellular cytotoxicity. European Journal of Cancer*,* 49 *(*15*),* 3353–3365; 23849826 10.1016/j.ejca.2013.06.011

[167] Jones, P. A., Baylin, S. B. (2007). The epigenomics of cancer. Cell*,* 128 *(*4*),* 683–692; 17320506 10.1016/j.cell.2007.01.029 PMC3894624

[168] Gangemi, R., Mirisola, V., Barisione, G., Fabbi, M., Brizzolara, A. et al. (2017). Mda-9/syntenin is expressed in uveal melanoma and correlates with metastatic progression. PLoS One*,* 7 *(*1*),* e29989. 10.1371/journal.pone.0029989 PMC3258266 22267972

[169] Vaisanen, A., Kallioinen, M., von Dickhoff, K., Laatikainen, L., Hoyhtya, M. et al. (1999). Matrix metalloproteinase-2 (MMP-2) immunoreactive protein--a new prognostic marker in uveal melanoma? The Journal of Pathology*,* 188 *(*1*),* 56–62; 10398141 10.1002/(SICI)1096-9896(199905)188:1<56::AID-PATH304>3.0.CO;2-B

[170] El-Shabrawi, Y., Ardjomand, N., Radner, H., Ardjomand, N. (2001). MMP-9 is predominantly expressed in epithelioid and not spindle cell uveal melanoma. The Journal of Pathology*,* 194 *(*2*),* 201–206; 11400149 10.1002/1096-9896(200106)194:2<201::AID-PATH840>3.0.CO;2-O

[171] Bi, M. C., Hose, N., Xu, C. L., Zhang, C., Sassoon, J. et al. (2016). Nonlethal levels of zeaxanthin inhibit cell migration, invasion, and secretion of MMP-2 via NF-κB pathway in cultured human uveal melanoma cells. Journal of Ophthalmology*,* 2016*,* 8734309; 26942004 10.1155/2016/8734309 PMC4749803

[172] Gardner, F. P., Serie, D. J., Salomao, D. R., Wu, K. J., Markovic, S. N. et al. (2014). c-MET expression in primary and liver metastases in uveal melanoma. Melanoma Research*,* 24 *(*6*),* 617–620; 25211165 10.1097/CMR.0000000000000118

[173] Surriga, O., Rajasekhar, V. K., Ambrosini, G., Dogan, Y., Huang, R. et al. (2013). Crizotinib, a c-Met inhibitor, prevents metastasis in a metastatic uveal melanoma model. Molecular Cancer Therapeutics*,* 12 *(*12*),* 2817–2826; 24140933 10.1158/1535-7163.MCT-13-0499

[174] El Filali, M., Van der Velden, PA., Luyten, GPM., Jager, MJ. (2012). Anti-angiogenic therapy in uveal melanoma. Developments in Ophthalmology*,* 49*,* 117–136; 22042017 10.1159/000329591

[175] Missotten, G. S., Notting, I. C., Schlingemann, R. O., Zijlmans, H. J., Lau, C. et al. (2006). Vascular endothelial growth factor a in eyes with uveal melanoma. Archives of Ophthalmology*,* 124 *(*10*),* 1428–1434; 17030710 10.1001/archopht.124.10.1428

[176] El Filali, M., Missotten, GS., Maat, W., Ly, LV., Luyten, GP. et al. (2010). Regulation of VEGF-A in uveal melanoma. Investigative Ophthalmology & Visual Science*,* 51 *(*5*),* 2329–2337. 20042655 10.1167/iovs.09-4739

[177] Francis, J. H., Kim, J., Lin, A., Folberg, R., Iyer, S. et al. (2017). Growth of uveal melanoma following intravitreal bevacizumab. Ocular Oncology and Pathology*,* 3 *(*2*),* 117–121; 28868282 10.1159/000450859 PMC5566762

[178] Tarhini, A. A., Frankel, P., Margolin, K. A., Christensen, S., Ruel, C. et al. (2011). Aflibercept (VEGF Trap) in inoperable stage III or stage iv melanoma of cutaneous or uveal origin. Clinical Cancer Research*,* 17 *(*20*),* 6574–6581; 21880788 10.1158/1078-0432.CCR-11-1463 PMC3196047

[179] Lattanzio, L., Tonissi, F., Torta, I., Gianello, L., Russi, E. et al. (2013). Role of IL-8 induced angiogenesis in uveal melanoma. Investigational New Drugs*,* 31 *(*5*),* 1107–1114; 23912257 10.1007/s10637-013-0005-1

[180] Bollag, G., Hirth, P., Tsai, J., Zhang, J., Ibrahim, P. N. et al. (2010). Clinical efficacy of a RAF inhibitor needs broad target blockade in BRAF-mutant melanoma. Nature*,* 467 *(*7315*),* 596–599; 20823850 10.1038/nature09454 PMC2948082

[181] Sosman, J. A., Kim, K. B., Schuchter, L., Gonzalez, R., Pavlick, A. C. et al. (2012). Survival in BRAF V600-mutant advanced melanoma treated with vemurafenib. The New England Journal of Medicine*,* 366 *(*8*),* 707–714; 22356324 10.1056/NEJMoa1112302 PMC3724515

[182] Chapman, P. B., Hauschild, A., Robert, C., Haanen, J. B., Ascierto, P. et al. (2011). Improved survival with vemurafenib in melanoma with BRAF V600E mutation. The New England Journal of Medicine*,* 364 *(*26*),* 2507–2516. 10.1056/NEJMoa1103782; 21639808 PMC3549296

[183] Shi, H., Hugo, W., Kong, X., Hong, A., Koya, R. C. et al. (2014). Acquired resistance and clonal evolution in melanoma during BRAF inhibitor therapy. Cancer Discovery*,* 4 *(*1*),* 80–93; 24265155 10.1158/2159-8290.CD-13-0642 PMC3936420

[184] Flaherty, K. T., Robert, C., Hersey, P., Nathan, P., Garbe, C. et al. (2012). Improved survival with MEK inhibition in BRAF-mutated melanoma. The New England Journal of Medicine*,* 367 *(*2*),* 107–114; 22663011 10.1056/NEJMoa1203421

[185] Long, G. V., Stroyakovskiy, D., Gogas, H., Levchenko, E., de Braud, F., et al. (2014). Combined BRAF and MEK inhibition versus BRAF inhibition alone in melanoma. The New England Journal of Medicine*,* 371 *(*20*),* 1877–1888; 25265492 10.1056/NEJMoa1406037

[186] Demirci, H., Demirci, F. Y., Ciftci, S., Elner, V. M., Wu, Y. M. et al. (2019). Integrative exome and transcriptome analysis of conjunctival melanoma and its potential application for personalized therapy. JAMA Ophthalmology*,* 137 *(*12*),* 1444–1448; 31647501 10.1001/jamaophthalmol.2019.4237 PMC6813594

[187] Pinto Torres, S., Andre, T., Gouveia, E., Costa, L., Passos, MJ. (2017). Systemic treatment of metastatic conjunctival melanoma. Case Reports in Oncological Medicine*,* 2017*,* 4623964; 29214089 10.1155/2017/4623964 PMC5682064

[188] Kiyohara, T., Tanimura, H., Miyamoto, M., Shijimaya, T., Nagano, N. et al. (2020). Two cases of BRAF-mutated, bulbar conjunctival melanoma, and review of the published literature. Clinical and Experimental Dermatology*,* 45 *(*2*),* 207–211; 31361915 10.1111/ced.14060

[189] Rossi, E., Maiorano, B. A., Pagliara, M. M., Sammarco, M. G., Dosa, T. et al. (2019). Dabrafenib and Trametinib in BRAF mutant metastatic conjunctival melanoma. Frontiers in Oncology*,* 9*,* 232; 31024839 10.3389/fonc.2019.00232 PMC6460374

[190] Larkin, J., Del Vecchio, M., Ascierto, P. A., Krajsova, I., Schachter, J. et al. (2014). Vemurafenib in patients with BRAF(V600) mutated metastatic melanoma: An open-label, multicentre, safety study. The Lancet Oncology*,* 15 *(*4*),* 436–444; 24582505 10.1016/S1470-2045(14)70051-8

[191] Welsh, S. J., Corrie, P. G. (2015). Management of BRAF and MEK inhibitor toxicities in patients with metastatic melanoma. Therapeutic Advances in Medical Oncology*,* 7 *(*2*),* 122–136; 25755684 10.1177/1758834014566428 PMC4346212

